# *Two out of Three Musketeers Fight against Cancer*: Synthesis, Physicochemical, and Biological Properties of Phosphino Cu^I^, Ru^II^, Ir^III^ Complexes

**DOI:** 10.3390/ph15020169

**Published:** 2022-01-29

**Authors:** Urszula K. Komarnicka, Alessandro Niorettini, Sandra Kozieł, Barbara Pucelik, Agata Barzowska, Daria Wojtala, Aleksandra Ziółkowska, Monika Lesiów, Agnieszka Kyzioł, Stefano Caramori, Marina Porchia, Alina Bieńko

**Affiliations:** 1 Faculty of Chemistry, University of Wroclaw, Joliot-Curie 14, 50-383 Wroclaw, Poland; sandra.koziel@chem.uni.wroc.pl (S.K.); daria.wojtala@chem.uni.wroc.pl (D.W.); ziolkowska.aleksandra@wp.pl (A.Z.); monika.lesiow@chem.uni.wroc.pl (M.L.); alina.bienko@chem.uni.wroc.pl (A.B.); 2 Department of Chemical, Pharmaceutical, and Agricultural Sciences, University of Ferrara, Via L. Borsari 46, 44121 Ferrara, Italy; alessandro.niorettini@unife.it (A.N.); cte@unife.it (S.C.); 3 Małopolska Centre of Biotechnology, Jagiellonian University, Gronostajowa 7A, 30-387 Krakow, Poland; barbara.pucelik@gmail.com (B.P.); agata.barzowska@doctoral.uj.edu.pl (A.B.); 4 Faculty of Chemistry, Jagiellonian University in Krakow, Gronostajowa 2, 30-387 Krakow, Poland; kyziol@chemia.uj.edu.pl; 5 ICMATE-CNR, Corso Stati Uniti, 4, 35127 Padova, Italy; marina.porchia@cnr.it

**Keywords:** iridium(III) complexes, ruthenium(II), cationic copper(I) complex, phosphines, lung cancer, DNA, reactive oxygen species, 3D spheroids

## Abstract

Two novel phosphine ligands, Ph_2_PCH_2_N(CH_2_CH_3_)_3_ (**1**) and Ph_2_PCH_2_N(CH_2_CH_2_CH_2_CH_3_)_2_ (**2**), and six new metal (Cu(I), Ir(III) and Ru(II)) complexes with those ligands: iridium(III) complexes: Ir(η5-Cp*)Cl_2_(**1**) (**1a**), Ir(η5-Cp*)Cl_2_(**2**) (**2a**) (Cp*: Pentamethylcyclopentadienyl); ruthenium(II) complexes: Ru(η6-p-cymene)Cl_2_(**1**) (**1b**), Ru(η6-p-cymene)Cl_2_(**2**) (**2b**) and copper(I) complexes: [Cu(CH_3_CN)_2_(**1**)BF_4_] (**1c**), [Cu(CH_3_CN)_2_(**2**)BF_4_] (**2c**) were synthesized and characterized using elemental analysis, NMR spectroscopy, and ESI-MS spectrometry. Copper(I) complexes turned out to be highly unstable in the presence of atmospheric oxygen in contrast to ruthenium(II) and iridium(III) complexes. The studied Ru(II) and Ir(III) complexes exhibited promising cytotoxicity towards cancer cells in vitro with IC_50_ values significantly lower than that of the reference drug—cisplatin. Confocal microscopy analysis showed that Ru(II) and Ir(III) complexes effectively accumulate inside A549 cells with localization in cytoplasm and nuclei. A precise cytometric analysis provided clear evidence for the predominance of apoptosis in induced cell death. Furthermore, the complexes presumably induce the changes in the cell cycle leading to G2/M phase arrest in a dose-dependent manner. Gel electrophoresis experiments revealed that Ru(II) and Ir(III) inorganic compounds showed their unusual low genotoxicity towards plasmid DNA. Additionally, metal complexes were able to generate reactive oxygen species as a result of redox processes, proved by gel electrophoresis and cyclic voltamperometry. In vitro cytotoxicity assays were also carried out within multicellular tumor spheroids and efficient anticancer action on these 3D assemblies was demonstrated. It was proven that the hydrocarbon chain elongation of the phosphine ligand coordinated to the metal ions does not influence the cytotoxic effect of resulting complexes in contrast to metal ions type.

## 1. Introduction

When it comes to pharmaceutical approaches to anticancer therapy, it is impossible not to mention cisplatin, as for over forty years it has been the active principle of choice in the fight against these diseases [1,2,3]. However, on the one hand, medicine has adopted it as one of the pillars of cancer therapy, while on the other hand, its prolonged use has confirmed the numerous limitations and risks, which cannot be ignored, related to the serious immediate and long-term side effects [4]. Cisplatin, in this regard, is also known to develop resistance to prolonged care cycles, a characteristic that makes it particularly ineffective in the event of the manifestation of a relapsed clinical picture [5]. Thanks to the experience gained, alternative strategies have been developed that have led to the synthesis of numerous organometallic platinum-based compounds such as carboplatin and nedaplatin, with the aim of developing selectively toxic molecules towards cancer cells, hoping to generate an efficient bio-distribution and an inability to develop resistance conditions [6]. For this purpose, the correlation between the activity of the metal and its chemical environment is fundamental, in fact, the possibility of using a wide range of ligands paves the way for the study of new and different mechanisms of action, which is capable of limiting the general toxicity associated with these drugs while, in the meantime, maximizing the efficiency of cellular uptake [7,8,9,10,11]. In recent years, the research has underlined how a synthetic approach of this type allows great progress in the synthesis and optimization of competitive chemotherapy; however, the intrinsic reactivity and biological characteristics of platinum make it difficult to overcome many important therapeutic obstacles and the consequent achievement of an ideal pharmacology. Due to this fact, the use of alternative metals is becoming a valid possible answer to the problem [5,12,13,14,15,16,17,18].

**Scheme 1:**
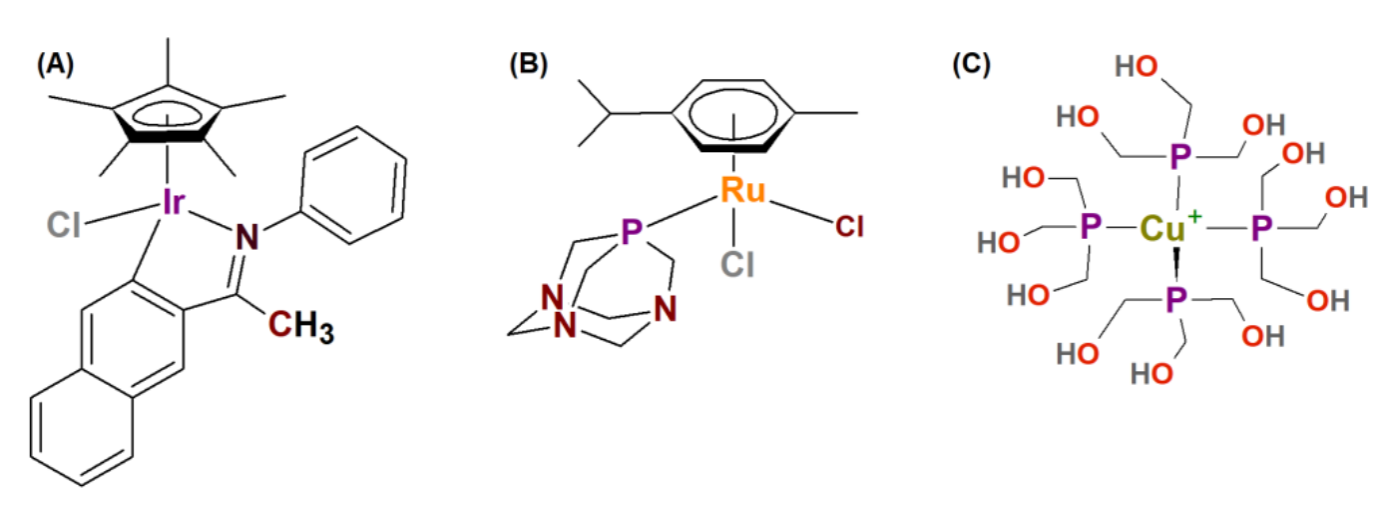
Schematic view of the (**A**) half-sandwich” Schiff-base Ir(III); (**B**) RAPTA-C; (**C**) [Cu(tris(hydroxymethyl)phosphine)_4_][PF_6_].

In relation to this overview, this work aims mainly to explore and understand the synergistic activity of phosphine ligands and copper, ruthenium or iridium ions, complexes with those metal ions are already known for their intrinsic antibacterial [19,20,21,22,23,24,25], antiviral [26,27,28,29], antifungal [30,31], and anti-inflammatory [32,33] characteristics (Scheme 1). Thanks to a wide range of coordination numbers, redox states, and possible geometries, inorganic compounds seem to possess a remarkable reactivity. In particular, a systematic study on synthesis and characterization of different metallic complexes in association with phosphine ligands was carried out, which are promising candidates for this purpose as their cytotoxicity towards tumor cells is already known [19,34,35,36,37,38,39,40,41,42,43].

In our work, we have synthesized two new phosphine ligands by attachment diethylamine(HN(CH_2_CH_3_)_2_) or dibuthylamine(HN(CH_2_CH_2_CH_2_CH_3_)_2_) to (hydroxymethyl)diphenylophosphine (Ph_2_PCH_2_OH). Structures of those ligands differ in the length of hydrocarbon chain. Interestingly, Mashid Kantoury with his team synthesized and studied the biological activity of three organometallic Pt(II) complexes with the general formula [Pt(phen)(L)]NO_3_, where phen–phenanthroline, L = methylglycine, amylglycine, and isopentylglycine. The differences between these three complexes are in the length of the hydrocarbon chain in ligands. Based on cytotoxic studies on the human breast cancer cell line (MDA-MB 231), the team managed to prove that, with an abridgment of the hydrocarbon chain in ligands, the cytotoxic activity of the resulting complexes increased [44]. Moreover, Paltan Laha with co-authors came to the same conclusion while investigating several mononuclear cyclometalatediridium(III) complexes towards the human breast cancer cell line (MCF-7) [45]. On the other hand, Swaminathan and her team came to a different conclusion after studying six novel Ru(II)-p-cymene complexes with various acylthiourea ligands against A549 and A549cisR cancer cell lines, as in this case—longer hydrocarbon chain improves the cytotoxicity [46]. Furthermore, in the literature, the copper(II) complexes with phen and alkyl chains with different lengths as ligands show the same pattern [47]. Interestingly, Ronald Gust and co-workers showed that cytotoxic activity of resulting compounds not always depend on hydrocarbon chain length. They proved that elongation of the spacer by one methylene group of Zeise’s salt derivatives increased their cytotoxicity, but further elongation slightly reduced the activity resulting compounds [48].

The correlation between the role of the metal and its chemical environment has therefore led to the synthesis of compounds with interesting pharmacokinetics; endowed with several biologically active functional structures capable of directing different mechanisms of action. The properties manifested by these compounds and, in particular by ruthenium and iridium, suggest a feasible future applicability. Therefore, this work shall be a useful tool for understanding the mechanisms of distribution, metabolization, and the role of these molecules in treating cancer pathologies, as a starting point for the optimization of new and more efficient drugs.

## 2. Results and Discussion

### 2.1. Synthesis

**Scheme 2:**
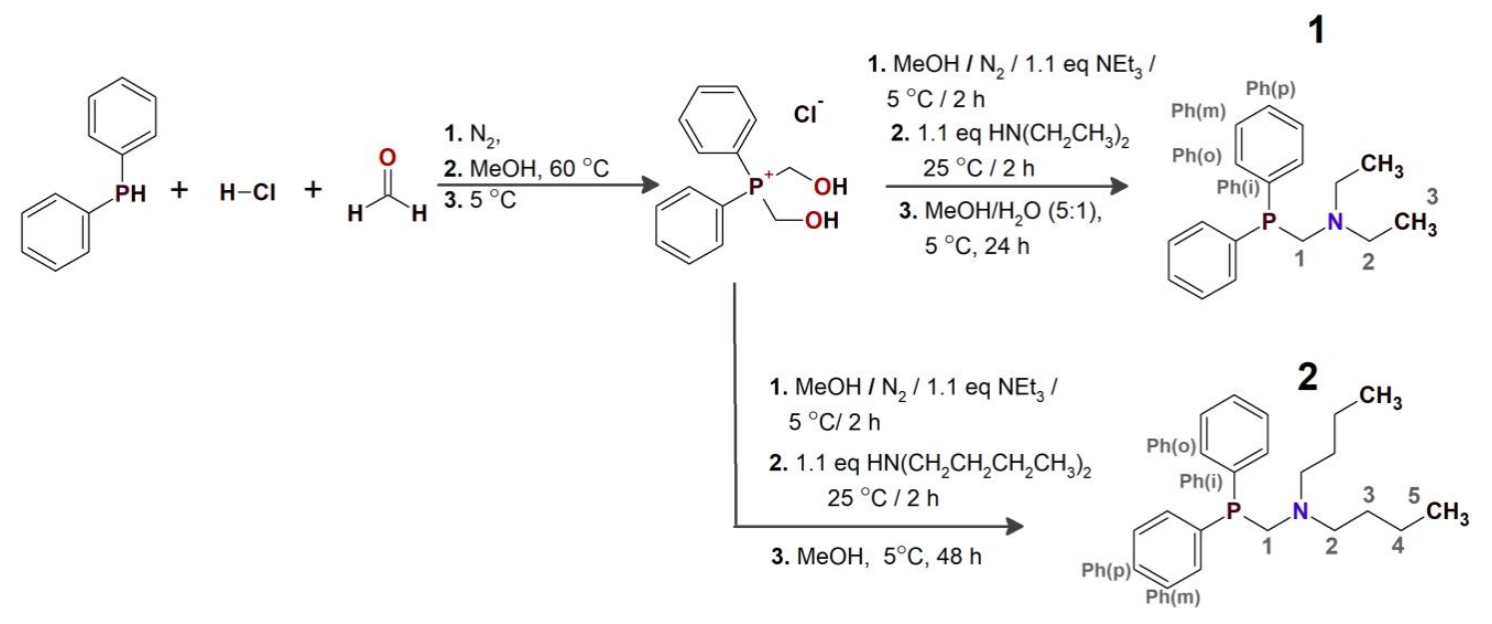
Schematic view of the phosphine (**1, 2**) and synthetic routes.

**Scheme 3:**
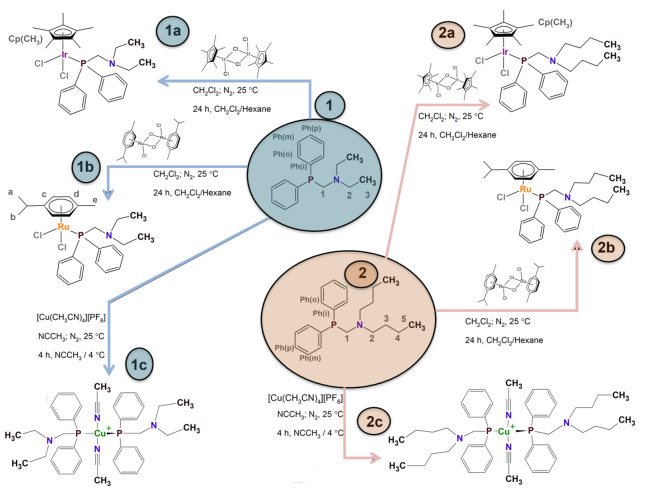
Schematic view of the Ir(III), Ru(II), Cu(I) complexes and synthetic routes.

All syntheses were carried out under an atmosphere of dry oxygen-free dinitrogen, using standard Schlenk techniques or a glove box. The ligands Ph_2_PCH_2_(CH_2_CH_3_)_2_ (**1**) and Ph_2_PCH_2_(CH_2_CH_2_CH_2_CH_3_)_2_ (**2**) (Scheme 2) are extremely sensitive and all reaction were performed in nitrogen atmosphere. Synthetic routes of iridium(III) complexes: Ir(η5-Cp*)Cl_2_Ph_2_PCH_2_(CH_2_CH_3_)_2_ (**1a**), Ir(η5-Cp*)Cl_2_Ph_2_PCH_2_(CH_2_CH_2_CH_2_CH_3_)_2_ (**2a**) (Cp*: pentamethylcyclopentadienyl); ruthenium(II) complexes: Ru(η6-p-cymene)Cl_2_ Ph_2_PCH_2_(CH_2_CH_3_)_2_ (**1b**), Ru(η6-p-cymene)Cl_2_Ph_2_PCH_2_(CH_2_CH_2_CH_2_CH_3_)_2_ (**2b**) and copper(I) complexes: [Cu(CH_3_CN)_2_(Ph_2_PCH_2_(CH_2_CH_3_)_2_)_2_] (**1c**), [Cu(CH_3_CN)_2_(Ph_2_PCH_2_(CH_2_CH_3_CH_3_CH_3_)_2_)_2_] (**2c**) are presented in Scheme 3.

**Figure 1:**
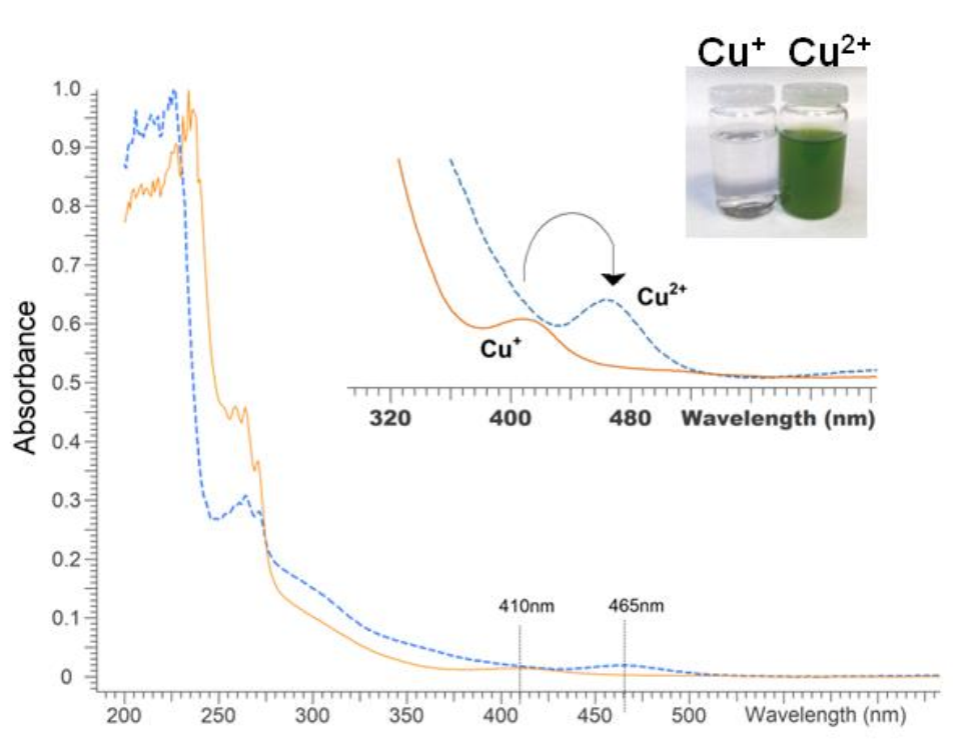
UV-Vis spectra for copper(I) complex **1c** registered after 0 h (dotted line, transparent colorless solution) and 12 h (solid line, green solution).

The copper(I) complexes were found to be non-air stable unlike Ru(II) and Ir(III) complexes. Oxidation of copper(I) to copper(II) was observed within 12 h using UV-Vis spectroscopy (Figure 1) that was additionally conformed by color of the copper(I) inorganic compound solution changing from transparent to green. Additionally, oxidation of phosphines ligands (**1**, **2**) was observed. It is worth mentioning that copper(I) ions can easily undergo oxidation processes [49]. The instability of copper(I) complexes prevented us from evaluating their biological activity.

All compounds are soluble in polar solvents such as dichloromethane and acetone, but insoluble in non-polar solvents such as pentane and hexane. The analytical data of all the copper(I), ruthenium(II), and iridium(III) complexes are in good agreement with the molecular formula proposed. The target complexes were characterized by NMR spectra, ESI(+)MS, and elemental analysis. These analytical and spectral data confirmed the stoichiometry of new complexes.

### 2.2. Structural Analysis of Cu(I), Ru(II), and Ir(III) Complexes

All phosphines Ph_2_PCH_2_N(CH_2_CH_3_)_2_ (**1**), Ph_2_PCH_2_N(CH_2_CH_2_CH_2_CH_3_)_2_ (**2**), and Ir(III) (**1a**, **2a**); Ru(II) (**1b**, **2b**) and Cu(I) (**1c**, **2c**) complexes were precisely characterized by 1D NMR techniques (^1^H NMR, ^31^P{^1^H} NMR) (Figure 2, Table S1, Figures S1–S9, Supplementary Material), and mass spectrometry (Figures S10–S23, Supplementary Material). The application of these techniques allowed determining the structures of the complexes in solution under atmospheric oxygen. 

**Figure 2:**
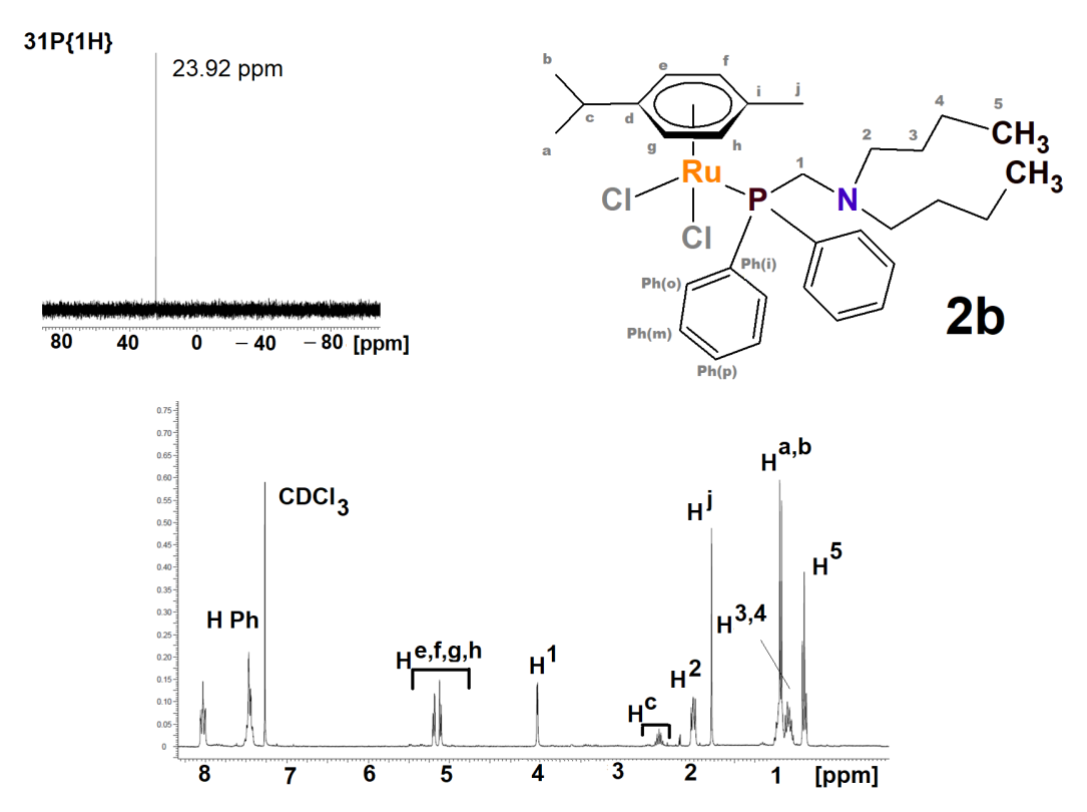
^31^P{^1^H} and ^1^H NMR spectra (298 K, DMSO) of the **2b**.

The ^31^P{^1^H} NMR analysis is a significant method for preliminary determination of sample purity and it was applied to verify if the product of synthesis is the desired one (Supplementary Materials, Table S1). The signal of uncoordinated phosphines is situated in the negative part of spectrum (**1**: −27.68 ppm and **2**: −26.72 ppm) and undergoes a downfield shift upon coordination (**1a**: −1.78 ppm; **1b**: 24.32 ppm; **1c**: −14.49 ppm, **2a**: −2.01 ppm, **2b**: 23.92 ppm (Figure 2) and **2c**: −13.07 ppm). Importantly, the absence of other signals in the spectra confirms that coordination compound is the only one product of synthesis, free both from uncoordinated phosphine, or other phosphine derivatives (e.g., phosphane oxides Supplementary Material, Table S1).

As expected, the biggest changes in the ^1^H NMR spectra (Supplementary Material, Table S1 and Figures S1–S9) upon the complexation process were observed for the atoms neighboring the coordinated metal ions: Cu(I), Ru(II), and Ir(III). Namely, almost all protons undergo an upfield shift (except signals of ^1^H for all complex–downfield shift) independently of the type of metal ion (Cu(I), Ir(III), and Ru(II)) and phosphine ligand (**1** or **2**). This might be related to the electron density increase around ligands caused by the formation of complex compounds. It is worth mentioning that after complexation, the variation of the chemical shifts of (PPh_2_CH_2_-) signals for Ir(III) and Ru(II) complexes compared to the free ligands is larger than those observed for Cu compounds. This means that both phosphine ligands are bounded via phosphorus atom much stronger to iridium and ruthenium ions compared to copper complexes and likely related to their instability. These observations are confirmed by UV-Vis data (described above) showing copper(I) to copper(II) oxidation within a few hours. 

According to the neutral character of Ir(III) complexes **1a** and **2a**, ESI-MS analysis confirmed the molecular ion peaks [M +H]^+^ in the positive ion modality at m/z 670.20 and 726.26, respectively. Less abundant peaks corresponding to [M–Cl]^+^ and [M–Cl–phosphine]^+^ ions were also recorded. In addition, loss of phosphine ligand and one chlorine ion gives the product ion at m/z 404.08, which rearranges with addition of an acetonitrile in both mass spectra of the complexes. Ru(II) complexes **1b** and **2b** molecular ion peaks [M + H]^+^ showed almost identical ESI(+) spectra giving the molecular ion peaks [M^+^H]^+^ at m/z 578.12 and 634.18, respectively. 

Regarding copper(I) compounds (**1c** and **2c**), the predominant peak in the ESI(+) full spectrum indicates that in solution the metal coordination environment of complexes rearranged due to the addition of ligand phosphine, with the simultaneous loss of the coordinated acetonitrile molecule. These results indicate the formation of mixed-ligand complexes. A peak due to the parent cluster ion of complex **1c** and **2c** was found at m/z 375.11 and 431.17, respectively. This could be identified with fragment ions of the general formula [Cu-phospine-CH_3_CN]^+^.

### 2.3. Oxidative Plasmid DNA Degradation

The ability of the phosphine ligands (**1**) and (**2**), and of the corresponding iridium(III) complexes (**1a**), (**2a**); ruthenium(II) complexes: (**1b**), (**2b**) as well as of dibuthylamine and diethylamine to induce single- or double-strand breaks in plasmid DNA was tested in the pBR322 plasmid (C = 0.5 mg/mL) (Supplementary Materials Figures S24–S27). DNA is a common molecular target of anticancer drugs. One of the ways of action of these substances is intercalation into DNA and degradation of its structure [50]. Understanding the mechanism of action of drugs plays a significant role in drug design due to the desire to obtain less toxic substances with greater potency [51]. 

The ability of studied compounds to induce single- and/or double-strand damage to DNA was tested using gel electrophoresis of pBR322 plasmid, which naturally occurs as a covalently closed superhelical form (form I). The process of DNA degradation can lead to the formation of both the relaxed/nicked form (form II) and linear form (form III) [52]. The degree of DNA degradation was determined in a wide range of concentrations (from 2 to 1000 µM). The obtained gel electrophoresis results show that regardless of used concentrations, none of the tested compounds caused DNA degradation. It indicates that studied compounds do not interact with DNA and their mechanism of action is different from cisplatin, the mode of action by interacting with the DNA represents one major reason for the severity of the side effects [53]. Due to the lack of DNA degradation, tested compounds can probably cause fewer side effects than cisplatin. This probably makes them a safe choice as potential cancer therapeutics. 

Hydrogen peroxide (H_2_O_2_) is a source of hydroxyl radicals and a strong DNA oxidant in the presence of transition metal ions and their complexes [54,55]. Gel electrophoresis can determine the ability of reactive oxygen species (ROS) to cause damage to plasmid DNA. In order to identify a given type of ROS formed by the complexes in the presence of H_2_O_2_, gel electrophoresis with the specific quenchers such as: DMSO (effective ^●^OH scavenger) [56] and NaN_3_ (effective ^1^O_2_ scavenger) [57] was performed. The studied complexes (**1a**, **1b**, **2b**, **2b**), in the presence of H_2_O_2_, are capable of causing DNA single-strand breaks and without causing double-strand breaks. Furthermore, the obtained results indicate that ruthenium complexes in the presence of hydrogen peroxide cause greater changes in the plasmid structure than iridium complexes (Supplementary Materials, Figure S27, lanes 7 and 15 compared to lanes 3 and 11). Partial protection against the formation of the nicked form of DNA was noticed after adding DMSO or NaN_3_ to the studied complexes in the presence of H_2_O_2_ (Supplementary Material, Figure S27.). This means that the tested ruthenium and iridium complexes are able to form moderate amounts of the hydroxyl radical and singlet oxygen.

**Figure 3:**
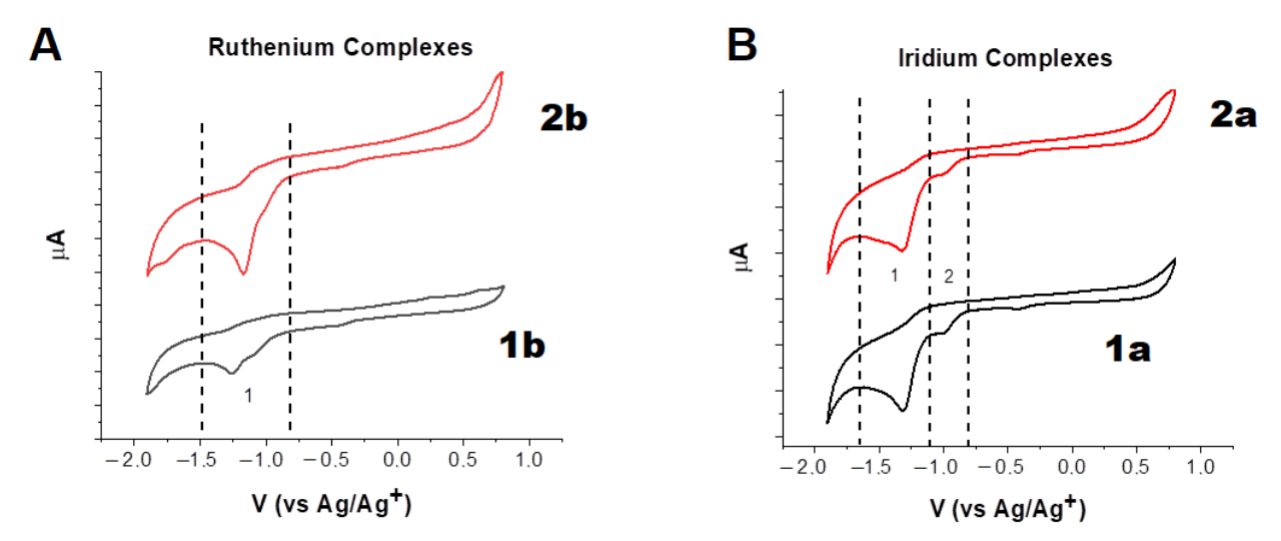
Cyclic voltammetries of (**A**) **2b** and **1b** in DMF + TBACl 0.1 M (vs. Ag/Ag^+^, scan rate 5mV/s) and (**B**) **2a** and **1a** in DMF + TBACl 0.1 M (vs. Ag/Ag^+^, scan rate 50 mV/s).

Furthermore, cyclic voltammetry (CV) was studied to precisely understand the re-dox activity of the studied complexes (i.e., ROS production, vide supra). Both iridium(III) and ruthenium(II) complexes manifest a similar electrochemical behavior, irrespective of the different substituents of the amine group (Figure 3). The cyclic voltammetry of all these mononuclear complexes, independently of the nature of the metal, reveal a main irreversible reductive wave centered at ca. −1.3 V vs. Ag/Ag+, which is the envelope of several irreversible processes beginning at ca. −0.8 V vs. Ag/Ag+, and are thus assigned to the phosphine ligand electro activity. In the case of the iridium complexes, and differently from the Ru(II) compounds herein considered, a comparatively small irreversible wave centered at about −1.0 V vs. Ag/Ag+, (peak 2 in Figure 1, left) separated well from the main peak at −1.3 V vs. Ag/Ag+ (peak 1 in Figure 1, left) is also observed.

### 2.4. In Vitro Anticancer Investigation

#### 2.4.1. Determination of IC_50_ and Partition Coefficients (LogP) Values

The lipophilicity of the potential drug has been shown to strongly influence the compounds’ efficacy. The logP value (oil/water partition coefficient) is well established to predict the biological activity of drugs and other biologically active compounds. In general, in the increase in lipophilicity, the biological activity of compounds increases because the affinity of substances with biological membranes and permeability is better, leading to better access to the site of action in the body. Nevertheless, this increase has to be controlled because high lipophilicity diminishes aqueous solubility, and consequently, the compound bioavailability. The more lipophilic drug may be may not be ideal for penetration through biological barriers as well as be accumulated in fatty tissue and difficult to excrete, leading to possible systemic toxicity [58,59]. Thus, the balance between hydrophilicity/lipophilicity of drug candidates is crucial in designing the therapeutic procedure with the selected drug candidate, its application route, and determines the interaction with the potential target [58,60,61].

With the increase in lipophilicity, the biological activity of compounds increases because the affinity of substances with biological membranes and permeability is better, leading to better access to the site of action in the body. At the same time, a further increase in lipophilicity causes an increase in the affinity for lipids and impedes the transport of molecules of the compound through the water phase, which results in its selective absorption in biological membranes.

The logP values for synthetized Ir(III) (**1a** and **2a**) and Ru(II) (**1b** and **2b**) complexes were determined and summarized in Table 1. The logP values of these complexes range between 3 and 4, which confirm their lipophilic nature. The longer the bicarbonate chain, the higher the logP value for the tested compounds. The effect of logP value on the bioavailability of cytostatic drugs can be seen in the performed biological studies and can diminish their in vitro availability.

Moreover, to investigate the activity of synthesized complexes as potential anticancer agents, their cytotoxicity toward a series of human cancer cell lines and a non-cancer cell line (HaCaT) was evaluated using the MTT assay. The IC_50_ values (the concentration at which 50% of cell growth is inhibited) for these compounds (after 24, 48, and 72 h) against a set of cells are summarized in Table 1 and Supplementary Materials Tables S2 and S3. In addition, we also determined the cytotoxic activity of free ligands toward selected cancer cells. As expected, free ligands show significantly lower cytotoxic activity than prepared metal complexes after all investigated incubation times (24, 48, and 72 h). However, this effect depends on the cell line. The complexes are about ten-fold more active than the phosphine ligands in A549 cells, while the ratio is about three to four in Du−145 cells, about two in MCF-7 cells, less than two in PANC-1 cells and about 0.6 in HaCaT cells. The best values of cytotoxic activity of compounds have been obtained after 72 h of cell incubation. Additionally, synthesized complexes had high sensitivity to A549 cells, so this cell line was selected as the research subject for antitumor activity.

#### 2.4.2. Cellular Accumulation

**Figure 4:**
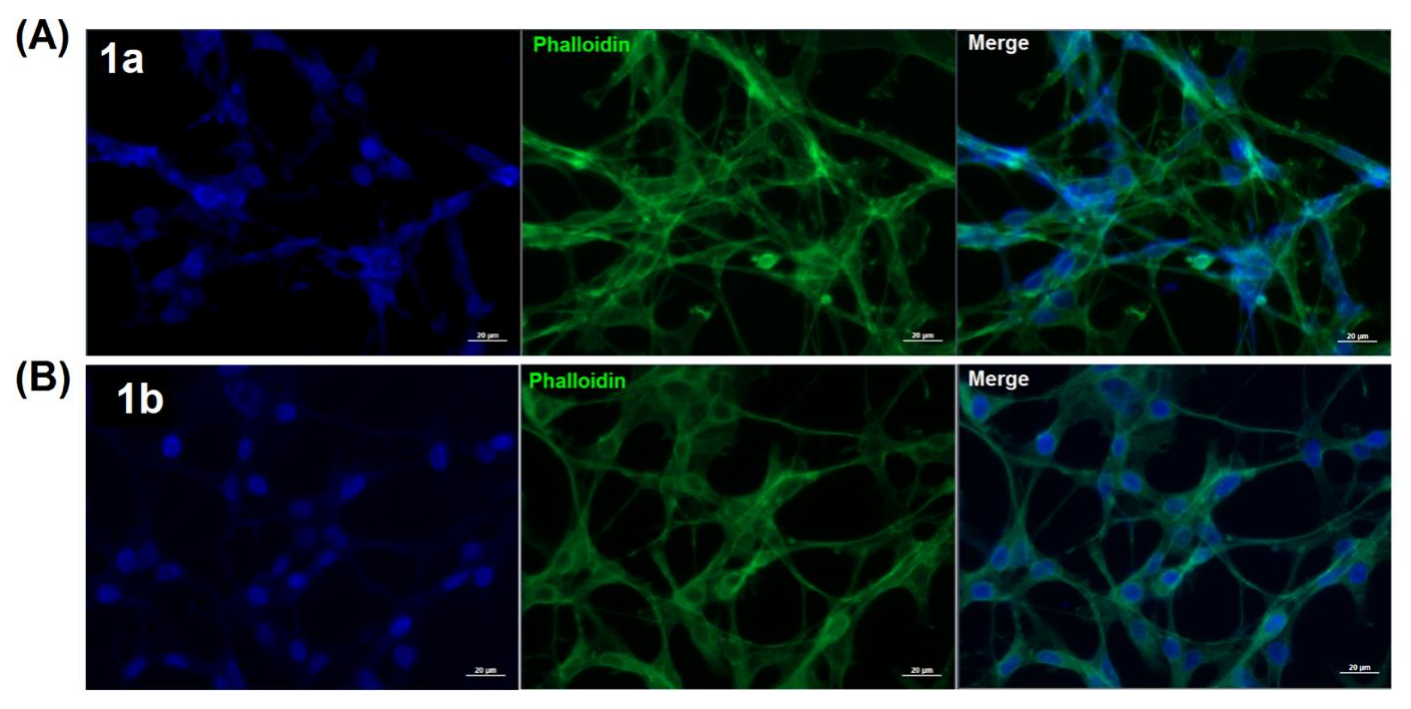
Accumulation of (**A**) Ir(III) (**1a**) and (**B**) Ru(II) (**1b**) in A549 cells. Green fluorescence–phalloidin, blue fluorescence-tested compounds.

It has been reported that the cellular uptake efficacy of a compound is affected by many factors, such as lipophilicity [62,63,64]. Synthesized complexes indicate blue fluorescence. Thus, their detection using fluorescence microscopy is possible. For this purpose, A549 (human lung adenocarcinoma epithelial) cells were incubated with Ir(III) complexes (5 μM) for 24 h and analyzed. Phalloidin was co-incubated with Ir(III) complexes to determine subcellular localization further. The complexes appeared as blue fluorescence, whereas cytoskeleton labeled with Phalloidin-FITC exhibited green fluorescence. As Figure 4 depicts, the blue fluorescence of complexes and the green fluorescence of phalloidin overlapped, indicating that the complexes were mainly accumulated in the nucleus and cytoplasm.

Fluorescence microscopic images show that both **1a** (5 μM) and **1b** (5 μM) can penetrate A549 cells (Figure 4). Nevertheless, in the case of **1a** Ir(III) complex, we observe the signal in whole (retain within the cytoplasm) cells with relative higher intensity in the nuclei (Figure 4A). Ru(II) complex **1b** localize mainly in nuclei, and the fluorescence in the cytoplasm is negligible (Figure 4B).

#### 2.4.3. Cell Death Mechanisms

Based on obtained results, it may be concluded that cell death mechanisms after treatment with **1a** differ from those induced by **1b**. Apoptosis induced by Ir(III) or Ru(II) complexes is not very obvious at a relatively lower concentration (1 μM). At a concentration of >5 μM, after the treatment with Ir(III) compounds, the percentage of late apoptotic cells increases from up to 60%. For Ru(II) compounds treated cells, the lower toxicity (more live cells) and mainly apoptotic cells are observed (up to 58%). In the case of **1a**, increasing concentration is accompanied by an increase of late apoptosis. The use of 5 and 10 μM induce more apoptosis than **1a** at 1 μM, and more cells remain in the late apoptosis stage than early apoptosis. It may suggest that early apoptotic cells can become late apoptotic cells quickly and the plasma membrane of these cells becomes permeabilized. After the treatment with the highest concentration (50 μM), ca. 15% of stained cells are necrotic cells. 

Analyzing cell death induced by **1b,** it can be seen that the analysis revealed that the treatment with each concentration resulted in more percentages of early apoptotic cells. After the treatment with a high concentration (50 μM), more late-apoptotic cells can be observed (30%). Nevertheless, there is significantly less population than after treatment with **1a**, where the late apoptosis is a major mechanism of death (up to 55% of AnnexinV-FITC-positive and PI-positive cells). Interestingly, almost no cells that died via the necrotic pathway may be detected. The increase in concentration accompanied by the extent of apoptosis seems to be favorable because agents that induce apoptosis directly would overcome many of the problems observed with existing drugs and, as a result, have great therapeutic potential. Apoptosis is preferable to necrosis since it is a particularly efficient mode of cell death that does not produce inflammation and damage to the surrounding normal tissue. Moreover, agents that induce apoptosis directly should be less mutagenic than existing drugs and, because they engage the program further downstream, be less prone to resistance [65]. 

**Figure 5:**
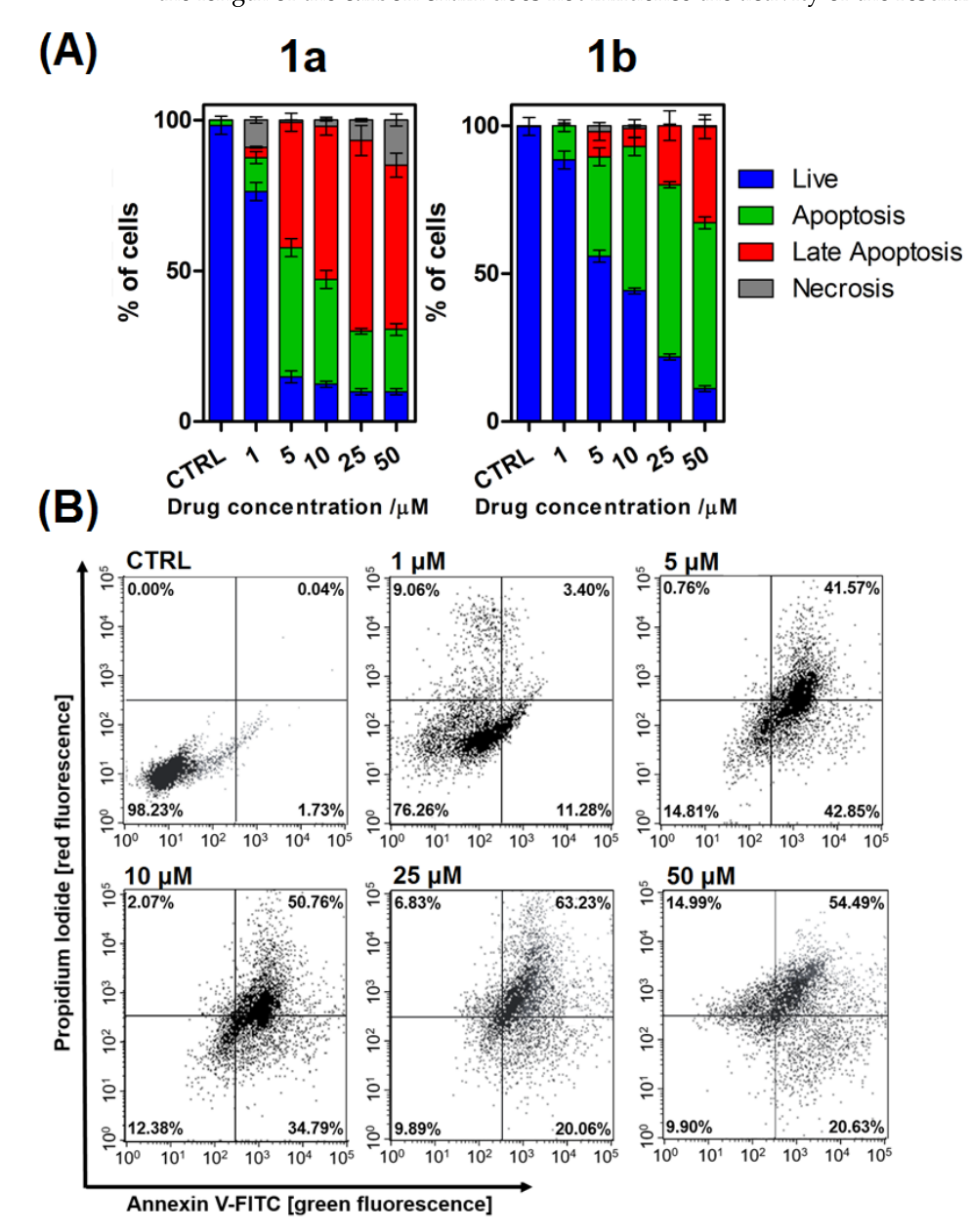
Cell death mechanisms (**A**) Annexin V-FITC/PI double staining of A549 cells treated with Ir(III) (**1a**) and Ru(II) (**1b**) at the indicated concentrations for 24 h.(**B**) Representative histograms showing cell death mechanisms in response to Ir(III) (**1a**) treatment.

The cell death induced by selected Ir(III) and Ru(II) complexes was assessed using Annexin V-FITC/PI double staining. Based on this assay the differentiation of the following cells population was possible: viable cells (Annexin-FITC negative, PI negative), apoptotic cells (Annexin-FITC positive, PI negative), late apoptotic cells (Annexin-FITC positive and PI positive), and necrotic cells (Annexin-FITC negative, PI positive). Treatment of A549 cells with Ir(III) or Ru(II) complexes leads to a dose-dependent increase in the percentage of cells in each phase (Figure 5). 

Apoptosis induced by Ir(III) or Ru(II) complexes is not very obvious at a relatively lower concentration (1 μM). At a concentration of >5 μM, after the treatment with Ir(III) compounds, the percentage of late apoptotic cells increases from up to 60%. For Ru(II) compounds treated cells, the lower toxicity (more live cells) and mainly apoptotic cells are observed (up to 58%). The results reveal that these complexes can induce apoptosis, displaying a similar apoptotic effect, which occurs earlier for Ir(III) compound. Interestingly, the length of the carbon chain does not influence the activity of the resulting complexes.

#### 2.4.4. Induction of Cell Cycle Arrest

The activity of studied complexes may also be associated with a disturbance of cell cycle progression, proliferation, and development. According to the literature, cell cycle arrest is commonly observed in many metal-based complex antitumor drugs [66]. Meanwhile, the anticancer activity of many anticancer complexes has been associated with cell cycle disturbances [67]. 

**Figure 6:**
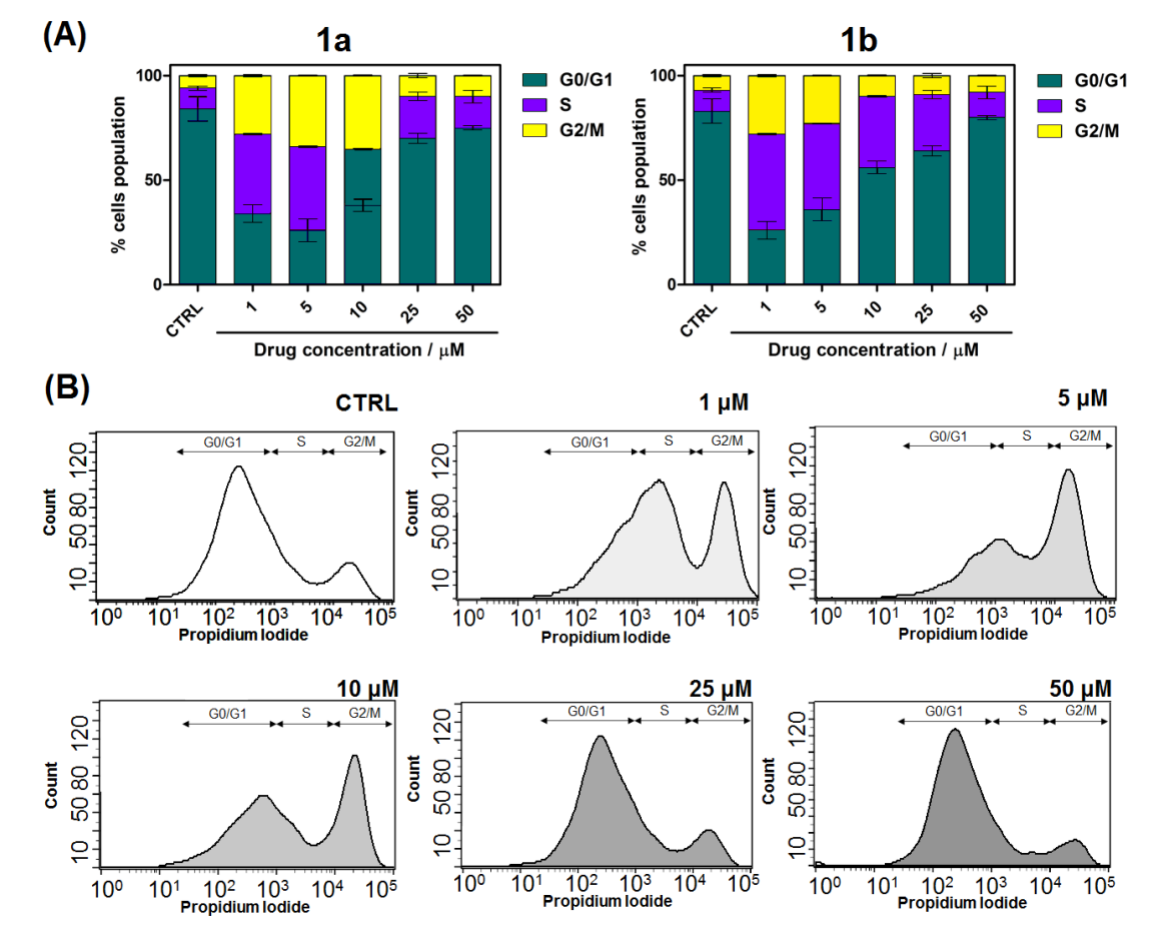
(**A**) Cell cycle analysis by PI staining after A549 cells were treated with Ir(III) and Ru(II) at the indicated concentrations for 24 h. (**B**) Representative histograms showing cell cycle changes in response to Ir(III) treatment.

Antitumor drugs induce cell cycle arrest by inhibiting DNA synthesis and enhancing DNA damage, ultimately leading cancer cells to apoptosis. We further studied the influence of Ir(III) and Ru(II) complexes on cell cycle distribution. Synchronized A549 cells were treated with Ir(III) (**1a**, **2a**) or Ru(II) (**1b**, **2b**) at various concentrations for 24 h. Cells were stained with propidium iodide (PI) and analyzed by flow cytometry. The result reveals that both compounds can elicit cell cycle arrest at the S stage at lower concentrations (up to 10 μM), Figure 6. An arrest in S-phase implies that the cell cannot duplicate its DNA. Other authors recently reported a similar effect for iridium(III)polypyridyl complexes [68,69]. Moreover, the percentage of the G2/M population also increases. However, higher doses (>10 μM) of Ir(III) complexes can enhance G0/G1-phase arrest. These results indicate that **1a** and **1b** elicit concentration-dependent cell cycle perturbations.

#### 2.4.5. Anticancer Activity in 3D Tumor Spheroids

**Figure 7:**
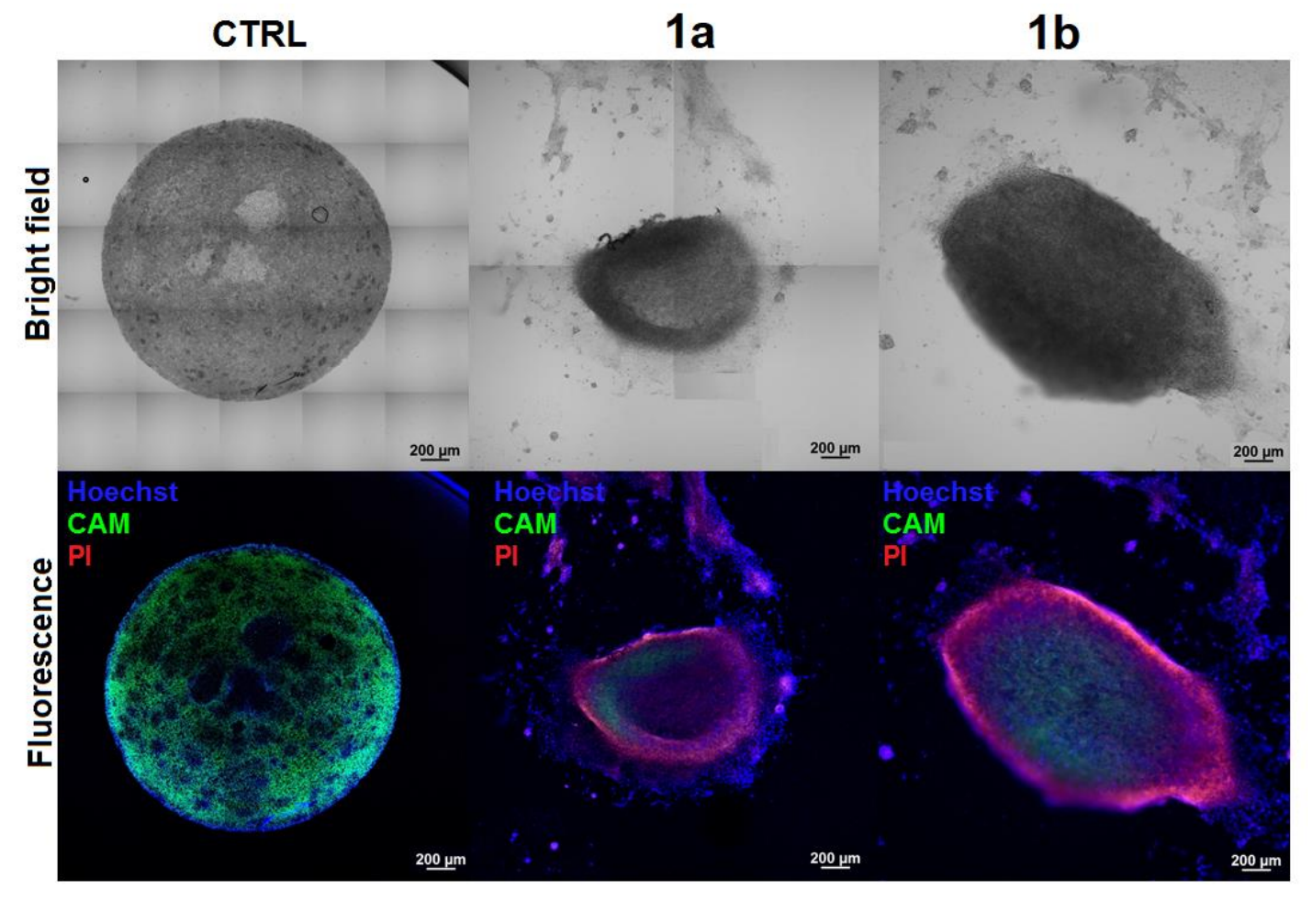
The cytotoxicity of Ir(III) **1a** and Ru(II) **1b** complexes against A549 efficacy against 3D tumor spheroid model.

Based on the promising results in the 2D cell culture, we also examined the anticancer activity of selected Ir(III) and Ru(II) complexes in the 3D A549 spheroid culture. The use of 3D spheroids culture provides a more accurate model of the in vivo conditions mimicking the solid tumor growth environment [70]. The therapeutic potential of the synthesized Ir(III) and Ru(II) compounds towards 3D spheroids was demonstrated by simultaneous Live/Dead fluorescence staining, providing the distribution of dead cells and cytotoxicity information (Figure 7). It may be seen that Ir(III) more significantly reduces the viability of 3D A549 spheroids, with huge morphology destruction. Nevertheless, the Ru(II) complex is active and leads to the destroyed spheroid structure. In contrast, the untreated spheroids retained their normal morphology and structure and dead cells were not observed.

## 3. Materials and Methods

### 3.1. Reagents

All syntheses and operations were carried out under an atmosphere of dry oxygen-free dinitrogen, using standard Schlenk techniques. Starting phosphine salt PPh_2_(CH_2_OH)_2_^+^Cl^−^ was synthesized according to literature procedures [54]. [Ir(η^5^-Cp*)Cl_2_]_2_ (>96%) was purchased from ACROS organics (Fisher Scientific; Geel–Belgium). [Ru(η6-p-cymene)Cl_2_]_2_ (1) (>98%), [Cu(CH_3_CN)_4_][BF_4_] (99.9%) and other reagents and solvent were purchased from Sigma Aldrich–Merck (Darmstadt–Germany). 

### 3.2. Methods

The NMR spectra were recorded on a Bruker AMX 500 spectrometer (at 25 °C) with traces of solvent as an internal reference for ^1^H (CDCl_3_: 7.26 ppm) and ^13^C{^1^H} spectra (CDCl_3_: 77.36 ppm) and 85% H_3_PO_4_ in H_2_O as an external standard for ^31^P{^1^H}. The signals in the spectra are defined as: s = singlet (*–strongly broadened signal), d = doublet, dd = doublet of doublets, t = triplet, and m = multiplet. Chemical shifts are reported in ppm and coupling constants are reported in Hz. Elemental analysis was carried out on a Vario EL3 CHN analyzer for C and H, and they were within 0.3% of the theoretical values. Absorption spectra were recorded on a Cary 50 Bio spectrophotometer (Varian Inc., Palo Alto, CA, USA) in the 800–200 nm range. Mass spectra were collected using a LCQ Fleet ion trap mass spectrometer (Thermo-Scientific, Waltham, Massachusetts, USA) equipped with an ESI source operating in the positive ion mode. Metal complexes were dissolved in chloroform giving ca. 2 × 10^−3^ M stock solutions. These solutions were subsequently diluted in methanol to obtain ca. 2 × 10^−5^ M solutions, which were directly infused into the ESI source via a syringe pump at a flow rate of 10 μL/min. The ions were produced using a spray voltage of 3.5 kV and the entrance capillary temperature was kept at 280 °C. Other instrumental parameters were automatically adjusted to optimize the signal to noise ratio.

### 3.3. Synthesis

#### 3.3.1. Preparation of Ph_2_PCH_2_N(CH_2_CH_3_)_2_ (**1**)

PPh_2_(CH_2_OH)_2_Cl (0.7434 g, 2.63 mmol) was dissolved in 30 mL of methanol and cooled down using a water bath (T = 5 °C). Then, a slight excess of NEt_3_ (triethylamine; 2.89 mmol) was added. The mixture was stirred, and with time, reached room temperature (RT). After 90 min, HN(CH_2_CH_3_)_2_ (0.1923 g, 2.63 mmol) was added dropwise into the mixture (RT). After 1 h of stirring, a clear solution was observed. The mixture was dried under reduced pressure for 3 h. White, crude product was dissolved in water (30 mL; the milky solution was observed) and extracted four times with CHCl_3_ (10 mL) using cannula. Chloroform phase was dried under pressure and white solid of (**1**) was formed. Ph_2_PCH_2_N(CH_2_CH_3_)_2_ was dissolved in methanol and kept at −18 °C at least for 24 h. After this time, white crystalline solid was formed. It is well soluble in CHCl_3_, DMSO, CH_2_Cl_2_, CH_3_CN, EtOH, MeOH, and moderately in H_2_O. Phosphine ligand is extremely sensitive to oxidation in solution. 

**Yield:** 88%, **Molar mass:** 271.34 g/mol. 

**Anal. Calc.** for C17H22NP: C, 75.25; H, 8.17; N, 5.16%. Found: C, 75.23; H, 8.18; N, 5.14%.

**^1^H NMR (300 MHz, CDCl_3_, 298 K):** δ 3.45 (H^1^, s), 2.79 (H^2^, q), 1.01 (H^3^, t).

**^31^P{^1^H}NMR (121 MHz, CDCl_3_, 298 K):** δ −27.68.

**ESI(+)MS in CH_3_CN, m/z (%):** 288.155 [M+H+OH]^+^; 272.156 [M+H]^+^; 199.069 [M-N(CH_2_CH_3_)_2_]^+^.

#### 3.3.2. Preparation of Ph_2_PCH_2_N(CH_2_CH_2_CH_2_CH_3_)_2_ (**2**)

PPh_2_(CH_2_OH)_2_Cl (0.6124 g, 2.17 mmol) was dissolved in 25 mL of methanol and cooled down using water bath (T = 5 °C). Then, a slight excess of NEt_3_ (triethylamine; 2.38 mmol) was added. Mixture was stirred and with time reached room temperature (RT). After 70 min, HN(CH_2_CH_2_CH_2_CH_3_)_2_ (0.2800 g, 2.17 mmol) was added drop-wise into the mixture (RT). After 2 h of stirring, clear solution was observed. Mixture was dried under reduced pressure. White, crude product was dissolved in deaerated water (30 mL; milky solution was observed) and extracted four times with deaerated CHCl_3_ (10 mL) using cannula. Chloroform phase was dried under pressure and white solid of (**2**) was formed. Ph_2_PCH_2_N(CH_2_CH_2_CH_2_CH_3_)_2_ was dissolved in acetonitrile and kept in −18 °C at least for 48 h. After this time, white crystalline solid were formed. It is well soluble in CHCl_3_, DMSO, CH_2_Cl_2_, CH_3_CN, EtOH, MeOH, and moderately in H_2_O. Phosphine ligand is extremely sensitive to oxidation in solution. 

**Yield**: 88%, **Molar mass:** 327.44 g/mol. 

**Anal. Calc.** for C21H30NP: C, 77.03; H, 9.24; C, 4.28%. Found: C, 77.02; H, 9.25; N, 4.26%.

**^1^H NMR (300 MHz, CDCl_3_, 298 K)**: δ 7.25–7.64 (H^Ph^), 3.48 (H^1^, s), 2.64 (H^2^, d), 1.34–1.50 (H^3^, m), 1.34–1.50 (H^4^, m), 0.89 (H^5^, t).

**^31^P{^1^H}NMR (121 MHz, CDCl_3_, 298 K)**: δ −26.72.

**ESI(+)MS in CH_3_CN, m/z (%)**: 465.346 [M+H+N(CH_2_CHCH_2_)_2_+CH_3_CN]^+^; 423.297 [M+N(CH_2_CHCH_2_)_2_]^+^; 344.218 [M+OH]^+^; 326.206 [M-H]^+^; 199.068 [M-N(CH_2_CH_2_CH_2_CH_3_)_2_]^+^.

#### 3.3.3. Preparation of Ir(η^5^-Cp*)Cl_2_Ph_2_PCH_2_N(CH_2_CH_3_)_2_ (**1a**)

Binuclear iridium complex of [Ir(η^5^-Cp*)Cl_2_]_2_ (0.2648 g, 0.332 mmol) was added to a solution of Ph_2_PCH_2_N(CH_2_CH_3_)_2_(0.1802 g, 0.664 mmol) in dichloromethane (25 mL). After a few minutes, the cloudy solution became clean and deep red. The mixture was stirred for 24 h and then the solvent was removed under vacuum. Crude **1a** was dissolved in the mixture of CH_2_Cl_2_ and hexane (V:V 1:1) and after 72 h in −5 °C crystalline red solid showed up. Ir(η^5^-Cp*)Cl_2_Ph_2_PCH_2_N(CH_2_CH_3_)_2_ is well soluble in CHCl_3_, CH_2_Cl_2_, methanol, DMSO, and mixture of H2O:DMSO (100:1).

**Yield:**78%, **Molar mass:**669.69 g/mol. 

**Anal. Calc.**for IrCl2C27H37PN: C, 48.42; H, 5.57; N, 2.09%. Found: C, 48.41; H, 5.59; N, 2.08%.

**^1^H NMR (300 MHz, CDCl_3_, 298 K):** δ 7.24–7.61 (H^Ph^, m), 4.01 (H^1^, s*), 2.25 (H^2^, s*), 0.72 (H^3^, s*), 1.47 (H^Cp*(CH3)^, s*).

^31^P{^1^H}NMR (121 MHz, CDCl_3_, 298 K): δ −1.78.

**ESI(+)MS in CH_3_CN, m/z (%):** 670.196 [M+H]^+^; 634.206 [M-Cl^-^]^+^; 404.080 [M+CH_2_CN-HCl-Ph_2_PCH_2_N(CH_2_CH_3_)_2_]^+^; 363.053 [M-Cl^-^-Ph_2_PCH_2_N(CH_2_CH_3_)_2_]^+^.

#### 3.3.4. Preparation of Ru(η^6^-p-cymene)Cl_2_Ph_2_PCH_2_N(CH_2_CH_3_)_2_(**1b**)

Binuclear ruthenium complex of [Ru(η^6^-p-cymene)Cl_2_]_2_ (0.2156 g, 0.3521 mmol) was added to a solution of Ph_2_PCH_2_N(CH_2_CH_3_)_2_ (0.1911 g, 0.7042 mmol) in dichloromethane (25 mL). After a few minutes, a cloudy solution became clean and orange. The mixture was stirred for 24 h and then the solvent was removed under vacuum. Crude **1b** was dissolved in mixture of CH_2_Cl_2_ and hexane (V:V 2:1) and kept in −18 °C for 72 h. After this time, orange crystalline solid showed up. Ru(η^6^-p-cymene)Cl_2_Ph_2_PCH_2_N(CH_2_CH_3_)_2_ is well soluble in CHCl_3_, CH_2_Cl_2_, methanol, DMSO, and mixture of H_2_O:DMSO (100:1). 

**Yield:** 79%, **Molar mass:** 577,53 g/mol. 

**Anal. Calc. for RuPNC27H36Cl2**: C, 56.15; H, 6.29; N, 2.42%. Anal.found: C, 56.12; H, 6.31; N, 2.41%

**^1^H NMR (300 MHz, CDCl_3_, 298 K)**: δ 7.31–8.02 (H^Ph^, m), 3.89 (H^1^, s*), 2.19 (H^2^, s*), 1.31 (H^3^, s*), 0.84 (H^a,b^, d), 2.97 (6.80) (H^c^, spt), 5.37 (36.0; 7.0) (H^e,f,h,i^, dd), 1.74 (H^j^, s).

**^31^P{^1^H}NMR (121 MHz, CDCl_3_, 298 K)**: δ 24.32.

**ESI(+)MS in CH_3_CN, m/z (%)**: 578.116 [M+H]^+^; 542.132 [M-Cl^-^]^+^; 312.012 [M+CH_3_CN-HCl-Ph_2_PCH_2_N(CH_2_CH_2_CH_2_CH_3_)_2_]^+^.

#### 3.3.5. Preparation of [Cu(NCCH_3_)_2_(Ph_2_PCH_2_N(CH_2_CH_3_)_2_)_2_]^+^BF_4_^−^ (**1c**)

Starting copper(I) complex [Cu(CH_3_CN)_4_][BF_4_] (0.4125 g, 1.1067 mmol) was dissolved in 10 mL of deaerated acetonitrile and added to a solution of Ph_2_PCH_2_N(CH_2_CH_3_)_2_ (1.2012 g, 4.4269 mmol; dissolved in deaerated acetonitrile 10 mL). After a few minutes, a cloudy solution became clean and transparent. The mixture was stirred for 10 h and then solvent was concentrated under vacuum and kept in −18 °C for 48 h. After this time, white crystalline solid formed out. [Cu(NCCH_3_)_2_(Ph_2_PCH_2_(CH_2_CH_3_)_2_)_2_]^+^PF_6_^−^ is well soluble in CHCl_3_, CH_2_Cl_2_, methanol, DMSO, and mixture of H_2_O:DMSO (100:1). 

**Yield:** 67%, **Molar mass:** 775.129 g/mol. 

**Anal. Calc.** for CuP2N4C38H50PBF_4_: C, 58.88; H, 6.50; N, 7.23%. Anal.found: C, 58.87; H, 6.52; N, 7.21%

**^1^H NMR (300 MHz, CDCl_3_, 298 K):** δ 7.28–7.49 (H^Ph^), 3.49 (H^1^, s*), 2.20 (H^2^, s*), 0.89 (H^3^, s*), 2.03 (H^acetonitrille^, s).

**^31^P{^1^H}NMR (121 MHz, CDCl_3_, 298 K)**: δ −14.49.

**ESI(+)MS in CH_3_CN, m/z (%):** 775.275 [M]^+^; 621.233 [M+OH−2H-(CH_3_CN)_2_-BF_4_^-^]^+^; 605.240 [M-H-(CH_3_CN)_2_-BF_4_^-^]^+^; 375.109 [M-H-PPh_2_CH_2_N(CH_2_CH_3_)_2_-CH_3_CN-BF_4_^-^]^+^; 320.030 [M-PPh_2_CH_2_N(CH_2_CH_3_)_2_-CH_3_-(CH_3_CN)_2_-BF_4_^-^]^+^.

#### 3.3.6. Preparation of Ir(η^5^-Cp*)Cl_2_Ph_2_PCH_2_N(CH_2_CH_2_CH_2_CH_3_)_2_ (**2a**)

Binuclear iridium complex of [Ir(η^5^-Cp*)Cl_2_]_2_ (0.2648 g, 0.332 mmol) was added to a solution of Ph_2_PCH_2_N(CH_2_CH_3_)_2_ (0.1802 g, 0.664 mmol) in dichloromethane (25 mL). After a few minutes a cloudy solution became clean and deep red. The mixture was stirred for 24 h and then the solvent was removed under vacuum. Crude 1a was dissolved in the mixture of CH_2_Cl_2_ and hexane (V:V 1:1) and after 72 h in −5 °C, crystalline red solid showed up. Ir(η^5^-Cp*)Cl_2_Ph_2_PCH_2_N(CH_2_CH_3_)_2_ is well soluble in CHCl_3_, CH_2_Cl_2_, methanol, DMSO, and mixture of H_2_O:DMSO (100:1).

**Yield:** 78%, **Molar mass:** 725.79 g/mol. 

**Anal. Calc.**for IrCl2C31H45PN: C, 51.30; H, 6.25; N, 1.93%. Found: C, 51.29; H, 6.27; N, 1.91%.

**NMR (298 K, CDCl3): ^31^P{^1^H}** (ppm): −2.01; ^1^H (ppm): H^Ph^7.30–8.02; H^1^ 4.02 s; H^2^ 2.09 s; H^3^ 0.72–0.98 m; H^4^ 0.72–0.98 m; H^5^ 0.71 t; H^Cp*(CH3)^ 1.37 s.

**ESI(+)MS in CH_3_CN, m/z (%)**: 726.255 [M]^+^; 690.269 [M-Cl]^+^; 404.078 [M-HCl-Ph_2_PCH_2_N(CH_2_CH_2_CH_2_CH_3_)_2_+CH_2_CN]^+^; 363.051 [M-HCl-Ph_2_PCH_2_N(CH_2_CH_2_CH_2_CH_3_)_2_]^+^.

#### 3.3.7. Preparation of Ru(η^6^-p-cymene)Cl_2_Ph_2_PCH_2_N(CH_2_CH_2_CH_2_CH_3_)_2_ (2b)

Binuclear ruthenium complex of [Ru(η^6^-p-cymene)Cl_2_]_2_ (0.2156 g, 0.3521 mmol) was added to a solution of Ph_2_PCH_2_N(CH_2_CH_3_)_2_ (0.1911 g, 0.7042 mmol) in dichloromethane (25 mL). After a few minutes, a cloudy solution became clean and orange. The mixture was stirred for 24 h and then the solvent was removed under vacuum. Crude 1b was dissolved in mixture of CH_2_Cl_2_ and hexane (V:V 2:1) and kept at −18 °C for 72 h. After this time, orange crystalline solid showed up. Ru(η^6^-p-cymene)Cl_2_Ph_2_PNCH_2_(CH_2_CH_3_)_2_ is well soluble in CHCl_3_, CH_2_Cl_2_, methanol, DMSO, and mixture of H_2_O:DMSO (100:1). 

**Yield**: 84%, **Molar mass**: 633,64 g/mol. 

**Anal. Calc.** for RuPNC31H44Cl2: C, 58.76; H, 6.99; N, 2.21%. Anal. found: C, 58.75; H, 7.01; N, 2.20%

**^1^H NMR (300 MHz, CDCl_3_, 298 K):** δ 7.41–8.04 (H^Ph^), 4.01 (H^1^, s), 1.98 (H^2^, s*), 0.70–0.89 (H^3^, m), 0.70–0.89 (H^4^, m), 0.68 (H^5^, t), 1.08 (H^a,b^, d), 2.51 (H^c^, spt), 5.15 (H^e,f,h,i^, dd), 1.75 (H^j^, s).

**^31^P{^1^H}NMR (121 MHz, CDCl_3_, 298 K):** δ 23.92.

**ESI(+)MS in CH_3_CN, m/z (%):** 634.177 [M+H]^+^; 598.195 [M-HCl]^+^; 312.013 [M+CH_3_CN+H-Cl^-^-Ph_2_PCH_2_N(CH_2_CH_2_CH_2_CH_3_)_2_]^+^.

#### 3.3.8. Preparation of [Cu(NCCH_3_)_2_(Ph_2_PCH_2_N(CH_2_CH_2_CH_2_CH_3_)_2_)_2_]^+^BF_4_^−^ (**2c**)

Starting copper(I) complex [Cu(CH_3_CN)_4_][BF_4_] (0.4125 g, 1.1067 mmol) was dissolved in 10 mL of deaerated acetonitrile and added to a solution of Ph_2_PCH_2_N(CH_2_CH_3_)_2_ (1.2012 g, 4.4269 mmol; dissolved in deaerated acetonitrile 10 mL). After a few minutes, a cloudy solution became clean and transparent. The mixture was stirred for 10 h and then solvent was concentrated under vacuum and kept at −18 °C for 48 h. After this time, white crystalline solid formed out. [Cu(NCCH_3_)_2_(Ph_2_PCH_2_N(CH_2_CH_3_)_2_)_2_]^+^BF_4_^−^ is well soluble in CHCl_3_, CH_2_Cl_2_, methanol, DMSO, and mixture of H_2_O:DMSO (100:1).

Yield: 73%, Molar mass: 827.203 g/mol. 

Anal. Calc. for CuP2N4C42H54BF_4_: C, 60.98; H, 6.58; N, 6.77%. Anal. found: C, 60.97; H, 6.60; N, 6.75%. 

^1^H NMR (300 MHz, CDCl_3_, 298 K): δ 7.25–7.32 (H^Ph^), 3.62 (H^1^, s), 2.08 (H^2^, s), 0.82–1.24 (H^3^, m), 0.82–1.24 (H^4^, m), 0.76 (H^5^, t), 2.02 (H^acetonitrille^, s).

^31^P{^1^H}NMR (121 MHz, CDCl_3_, 298 K): δ −13.07.

ESI(+)MS in CH_3_CN, m/z (%): 733.363 [M+OH-2H-(CH_3_CN)_2_-BF_4_^-^]^+^; 717.368 [M-H-(CH_3_CN)_2_-BF_4_^-^]^+^; 431.173 [M-H-PPh_2_CH_2_N(CH_2_CH_2_CH_2_CH_3_)_2_-CH_3_CN-BF_4_^-^]^+^; 328.225 [M-Cu-PPh_2_CH_2_N(CH_2_CH_2_CH_2_CH_3_)_2_-(CH_3_CN)_2_-BF_4_^-^]^+^.

### 3.4. Electrochemical Measurements

The Ru(II) and Ir(III) (C = 0.5 mM) complexes synthetized, respectively, with two different phosphinic ligands functionalized with ethylamine and butylamine were characterized by cyclic voltammetry analysis using a PGSTAT302N potentiostat-galvanostat, in order to delineate their characteristic redox profile. The analysis was carried out on a single compartment cell with a classical three electrode configuration, a glassy carbon as working, an Ag/Ag^+^ electrode as a reference, and platinum thick wire as counter. An electrolytic solution based on DMF + TBACl 0.1 M was adopted with an analyte concentration of 0.5 mM, previously deoxynenated with nitrogen for 15 min before the start of the experiments and continuously for the entire duration of the measures to maintain a constant slight nitrogen back pressure in the cell headspace. Cyclic voltammetries were performed by scanning clockwise (oxidation first followed by reduction) the potentials included in the electrolyte stability window (identified from +0.8 to −1.9 V vs. Ag/Ag^+^) starting from the OCP, *open circuit potential*, confirmed at +0.2 V vs. Ag/Ag^+^. Each voltammetric profile was dominated by the presence of an intense broad peak present at negative potentials characteristic of the reduction of the metal, minor peaks often not clearly distinguishable from the main metal process were attributed to the activity of the ligand. Due to the high sensitivity to air and relative instability, it was not possible to carry out tests on the similar copper complexes, CuPPh_2_CH_2_N(CH_2_CH_2_CH_2_CH_3_)_2_, and CuPPh_2_CH_2_N(CH_2_CH_3_)_2_.

### 3.5. DNA Strand Break Analysis

The ability of **1**, **1a**, **1b**, **2**, **2a**, **2b**, and starting ligands to induce plasmid DNA damage was tested with the pBR322 plasmid (C = 0.5 mg/mL). All compounds were dissolved in DMF with/without hydrogen peroxide (H_2_O_2_, C = 50 μM), dimethyl sulfoxide (DMSO, C = 1.4 mM), or sodium azide (NaN_3_, C = 40 mM) [71]. After incubation for 1 h at 37 °C, reaction mixtures (16 μL) were mixed with 3 μL of loading buffer (bromophenol blue in 30% glycerol) and loaded on 1% agarose gels, containing EB, in TBE buffer (90 Mm Tris–borate, 20 mM EDTA, pH = 8.0). Gel electrophoresis was performed at a constant voltage of 120 V for 120 min. The gel was photographed and processed with a Digital Imaging System (Syngen Biotech, Wroclaw, Poland).

### 3.6. Partition Coefficient 

The partition coefficient was determined by the shake-flask method. A small amount of compound was dissolved in 5 mL PBS-saturated n-octanol. The sample was sonicated until all the compound was dissolved. Next, 5 mL of PBS saturated with n-octanol was added and the experiment proceeded as before. The resulting mixture was vortexed for 30 min in horizontal position. The sample was centrifuged for 2 min at 3700 rpm to obtain a phase separation. Next, 2 mL of each phase was taken. The next steps were a 5 min sonication and measurement of absorption spectra of the obtained solutions. The calibration curve was prepared in order to determine the concentration of the tested compounds in individual solutions. It was made using the absorption intensity of the compounds in series dilution solutions in PBS saturated with octanol or octanol saturated with PBS in the concentration range c = 1–100 nM.

### 3.7. Cell Cultures

MCF7 cell line (human breast adenocarcinoma, morphology: epithelial-like, ATCC: HTB-22) and A549 cell line (human lung adenocarcinoma, morphology: epithelial, ATCC: CCL-185); HaCaT (human keratinocyte) were cultured in Dulbecco’s Modified Eagle’s Medium (DMEM, Corning, Poland) with phenol red, supplemented with 10% fetal bovine serum (FBS) and with 1% streptomycin/penicillin. Du-145 cell line (human prostate carcinoma); derived from metastatic site: brain was cultured in minimum essential medium (MEM, Corning) with only 10% fetal bovine serum (FBS). Cultures were incubated at 37 °C under a humidified atmosphere containing 5% CO_2_. Cells were passaged using a solution containing 0.05% trypsin and 0.5 mM EDTA. All media and other ingredients were purchased from ALAB, Poland.

### 3.8. Cytotoxic Activity

Since most of the studied compounds are insoluble in aqueous media (Dulbecco’s Modified Eagle’s Medium or minimum essential medium (MEM, Corning), therefore, they needed to be pre-dissolved in DMSO for biological tests. DMSO concentration was kept below 1% in each tested concentration of each compound. Cytotoxicity was assessed by MTT assay performed according to the protocols described previously [52] in the following experimental approaches: 6 h + 24 h, 24 h + 24 h, and 72 h + 24 h. In brief, 5×10^3^ per well (0.2 mL) in the two first approaches, while 1×10^4^ cells in the case of longer incubation time were seeded in 96-well flat-bottom microtiter plate, were incubated with the tested complexes at various ranges of concentrations. After an appropriate incubation time of 6, 24, or 72 h, the cells were washed thee time with PBS and fresh medium was applied for a further 24 h in each approach. Various compound concentrations (dilutions in proper medium supplemented with 1% FBS) were tested in five replicates and repeated at least three times. Determined values of IC_50_ (concentration of a drug required to inhibit the growth of 50% of the cells) are given as mean + S.D. (Standard Deviation). IC_50_ values were determined, from the plots of cell viability in the presence of various concentrations of each compound by matching dose–response curves and using the Origin 9.0 software. Furthermore, post-treatment survival assessment of the treated cells was analyzed under an inverted fluorescence microscope (Olympus IC51, Japan) with an excitation filter 470/20 nm. For this, cells were stained with two versatile fluorescence dyes: fluorescein diacetate (FDA, 5 mg/mL) and propidium iodide (PI, 5 mg/mL) in standard conditions in the dark for 20 min. Before visualization, dyes were removed and cells were washed with PBS twice. 

### 3.9. Intracellular Accumulation

The intracellular accumulation of the compound was assessed in the A549 cell line. Before imaging, cells were seeded on microscopic slides at a density of 1×10^5^ cells and were kept at 37 °C with 5% CO_2_ humidified atmosphere for 24 h. After being washed with fresh medium, the cells were incubated with 5 μM solution of tested compound in cell medium for 24 h. Next, after being washed with HBSS, the cells were fixed for 10 min in 3.8% PFA and incubated with specific intracellular organelle probes: 200 nM Mito-Tracker green, 1 μg/mL Quinacrine, and 0.25 nM of PhalloidinAtto 488 (Molecular Probes, Invitrogen Life Technologies; Thermo Fisher Scientific, Waltham, MA, USA), diluted in HBSS buffer. After 30 min incubation, at 37 °C, in the dark, the cells were washed with HBSS two times, and the slide was transferred to the microscope stage and cells were visualized under a fluorescent Zeiss Axio Observer microscope. Registered images were analyzed with the Zeiss ZEN software.

### 3.10. Flow Cytometry

Cell death was quantified using the AnnexinV-FITC/PI double staining kit (Sigma Aldrich). The cells (1 × 10^5^ per well) were cultured overnight in 12-well plates. Then, cells were treated with tested compounds for 24 h. After this time, cells were passaged, collected by centrifugation, and washed twice in Hank’s Balanced Salt Solution (HBBS). The cells were then re-suspended in 500 μL binding buffer and stained with annexin V-FITC and PI according to the manufacturer’s instructions (Sigma Aldrich). Stained A549 cells were then examined using a Guava^®^ easyCyte™ flow cytometer equipped with a 488 nm laser. The obtained data were analyzed using InCyte software (MerckMillipore, Burlington, MA, USA).

The serum starvation protocol was used to synchronize cell cultures. A549 cells were seeded in a 12-well plate in a growth medium with 20% FBS overnight. Then, the cultures were washed by PBS and changed to a serum-free medium. After serum starvation for 24 h, cells were released into the cell cycle by the addition of serum and tested compounds solution for 24 h. The cells were then detached from the plate using trypsin. Then, after washing, they were incubated with 50 µg/mL propidium iodide for 15 min in the dark. Cells were analyzed with the Guava^®^ easyCyte™ flow cytometer equipped with a 488 nm laser. The obtained data were analyzed using InCyte Software (MerckMillipore, Burlington, MA, USA).

### 3.11. Three-Dimensional Culturing In Vitro

Three-dimensional culturing in vitro: For hanging drop formation, the lid from a tissue culture dish was removed and 5 × 10^5^ CT-26 or A549 cells in 10 μL drops were placed on the bottom of the lid. In each case, the cell suspension was homogeneous and did not contain aggregates, since it determines the size and uniformity of spheroids. Then, the lid was inverted onto the PBS-filled bottom chamber. The cells were cultured in incubators maintained at 37 °C with 5% CO_2_ under fully humidified conditions. Once sheets were formed, they were transferred to 96-well plates coated with Geltrex matrix and incubated with a complete growth medium until spheroids were created. The direct effect of compound toxicity was examined on spheroids derived from both CT-26 and A549 cell lines. Twenty-four hours after treatment, the spheroids were stained with Hoechst33342, calcein AM (CAM), and propidium iodide (PI) to estimate the live/dead cells population for one hour, washed, and visualized under a Zeiss LSM 880 confocal microscope (Carl Zeiss, Jena, Germany) with a 63× immersion objective. Images were analyzed by Zeiss ZEN Software.

## 4. Conclusions

Herein, we present for the first time two novel phosphine ligands, Ph_2_PCH_2_N(CH_2_CH_3_)_3_ (**1**) and Ph_2_PCH_2_N(CH_2_CH_2_CH_2_CH_3_)_2_ (**2**), and six new metal (Cu(I), Ir(III) and Ru(II)) complexes with those ligands: iridium(III) complexes: Ir(η^5^-Cp*)Cl_2_(**1**) (**1a**), Ir(η^5^-Cp*)Cl_2_(**2**) (**2a**); ruthenium(II) complexes: Ru(η^6^-p-cymene)Cl_2_(**1**) (**1b**), Ru(η^6^-p-cymene)Cl_2_(**2**) (**2b**) and copper(I) complexes: [Cu(CH_3_CN)_2_(**1**)BF_4_] (**1c**), [Cu(CH_3_CN)_2_(**2**)BF_4_] (**2c**). All compounds were precisely characterized by elemental analysis, selected spectroscopic methods (i.e., absorption and NMR), ESI-MS, and electrochemical techniques. It was proven that copper(I) compounds are not stable in the presence of atmospheric oxygen in contrast to iridium and ruthenium compounds. In addition, we have studied cytotoxic effects of Ru(III) and Ir(III) complexes *in vitro* towards selected cancer cell lines. We demonstrated in vitro that complexes are characterized by lower IC_50_ values than that of cisplatin. Furthermore, preliminary investigation on the elucidation of action of the selected compounds towards A549 lung cell line allowed us to formulate the following general conclusions: 

(i) Complexes can penetrate into A549 cells.

(ii) Ru(II) and Ir(III) complexes can induce apoptosis, displaying the similar apoptotic effect, which occurs earlier for Ir(III) compound. 

(iii) Compounds can elicit cell cycle arrest at S stage at lower concentrations; however, higher doses (>10 μM) of Ir(III) complexes can enhance G0/G1-phase arrest. 

(iv) Redox potentials enable efficient ROS generation that causes plasmid DNA degradation, 

Finally, the therapeutic potential of the synthesized Ir(III) and Ru(II) compounds towards 3D spheroids was demonstrated by simultaneous in situ Live/Dead fluorescence staining, providing the spatial distribution of dead cells and cytotoxicity information. It was proven that Ir(III) more significantly reduces the viability of 3D A549 spheroids, with huge morphology destruction. Importantly, the length of the hydrocarbon chain of phosphine ligands does not influence the cytotoxic activity of resulting complexes. In vitro anticancer effect mostly depends on metal ion type. In our case, inorganic compounds with iridium seem to be much more effective towards cancer cells than ruthenium ones. 

## Figures and Tables

**Table 1:** IC_50_ (µM) values of the investigated complexes toward the selected cancer cell lines for 72 h and log*P_o/w_*. Data are given as mean ± SD of 3 independent experiments (*N* = 3).

	MCF-7	Du-145	A549	PANC-1	HaCaT	Experimental LogP
**dibutylamine**	>1000	>1000	>1000	>1000	>1000	nd
**diethylamine**	>1000	>1000	>1000	>1000	>1000	nd
**1**	192 ± 20	86 ± 6	183 ± 12	185 ± 12	490 ± 12	nd
**2**	285 ± 11	57 ± 4	158 ± 11	142 ± 20	448 ± 25	nd
**1a**	85 ± 8	19 ± 3	12 ± 3	91 ± 13	320 ± 19	3.03
**2a**	56 ± 6	13 ± 2	16 ± 3	85 ± 16	302 ± 22	4.0
**1b**	145 ± 15	23 ± 2	10 ± 1	128 ± 14	303 ± 20	3.1
**2b**	121 ± 20	17 ± 3	14 ± 2	112 ± 18	347 ± 25	3.84
**Cisplatin**	64 ± 4	50 ± 2	11 ± 3	79 ± 5	102 ± 2	nd.

nd–not determined.

## Data Availability

Data is contained within article and Supplementary Materials.

## Supplementary Materials

The following supporting information can be downloaded at: https://www.mdpi.com/article/10.3390/ph15020169/s1, Table S1 Cumulative NMR data for ligands and iridium-complexes.; Figure S1: ^1^H and ^31^P{^1^H} NMR spectra for 1 (298 K, CHCl_3_-d); Figure S2: ^1^H and ^31^P{^1^H} NMR spectra for **1a** (298 K, CHCl_3_-d); Figure S3: ^1^H and ^31^P{^1^H} NMR spectra for **1b** (298 K, CHCl_3_-d); Figure S4: ^1^H and ^31^P{^1^H} NMR spectra for **1c** (298 K, CHCl_3_-d); Figure S5: ^1^H and ^31^P{^1^H} NMR spectra for **2** (298 K, CHCl_3_-d); Figure S6: ^1^H and ^31^P{^1^H} NMR spectra for **2a** (298 K, CHCl_3_-d); Figure S8: ^1^H and ^31^P{^1^H} NMR spectra for **2b** (298 K, CHCl_3_-d); Figure S9: ^1^H and ^31^P{^1^H} NMR spectra for **2c** (298 K, CHCl_3_-d); Figure S10: Full ESI(+)MS spectrum of **1**.; Figure S11: Experimental and simulated ESI(+)MS spectrum of **1**.; Figure S12: Full ESI(+)MS spectrum of **2**.; Figure S13: Experimental and simulated ESI(+)MS spectrum of **2**.; Figure S14: Full ESI(+)MS spectrum of **1a**.; Figure S15: Experimental and simulated ESI(+)MS spectrum of **1a**.; Figure S16: Full ESI(+)MS spectrum of **1b**.; Figure S17: Experimental and simulated ESI(+)MS spectrum of **1b**.; Figure S18: Full ESI(+)MS spectrum of **1c**.; Figure S19: Experimental and simulated ESI(+)MS spectrum of **1b**.; Figure S20Full ESI(+)MS spectrum of **2a**.; Figure S19: Experimental and simulated ESI(+)MS spectrum of **2a**.; Figure S20: Full ESI(+)MS spectrum of **2b**.; Figure S21: Experimental and simulated ESI(+)MS spectrum of **2b**.; Figure S22: Full ESI(+)MS spectrum of **2c**.; Figure S23: Experimental and simulated ESI(+)MS spectrum of **2c**.; Figure S24: Agarose gel electrophoresis of pBR322 plasmid cleavage by diethylamine and dibuthylamine.; Figure S25: Agarose gel electrophoresis of pBR322 plasmid cleavage by phosphines; Figure S26: Agarose gel electrophoresis of pBR322 plasmid cleavage by Cu(I), Ru(II) and Ir(III) complexes.; Figure S27: Agarose gel electrophoresis of pBR322 plasmid cleavage by Ir-ethyl, Ru-ethyl, Ir-but and Ru-but complexes. Lanes: 1, plasmid−control; 2, plasmid + 50 μM Ir-ethyl; 3, plasmid + 50 mΜ Ir-ethyl + 50 μM H_2_O_2_; 4, plasmid + 50 μM Ir-ethyl + 50 μM H_2_O_2_ + 1.4 mM DMSO; 5, plasmid + 50 μM Ir-ethyl + 50 μM H_2_O_2_ + 40 mM M NaN_3_; 6, plasmid + 50 μM Ru-ethyl; 7, plasmid + 50 μM Ru-ethyl + 50 μM H_2_O_2_; 8, plasmid + 50 μM Ru-ethyl + 50 μM H_2_O_2_ + 1.4 mM DMSO; 9, plasmid + 50 μM Ru-ethyl + 50 μM H_2_O_2_ + 40 mM M NaN_3_; 10, plasmid + 50 μM Ir-but; 11, plasmid + 50 μM Ir-but + 50 μM H_2_O_2_; 12, plasmid + 50 μM Ir-but + 50 μM H_2_O_2_ + 1.4 mM DMSO; 13, plasmid + 50 μM Ir-but + 50 μM H_2_O_2_ + 40 mM NaN_3_; 14, plasmid + 50 μM Ru-but; 15, plasmid + 50 μM Ru-but + 50 μM H_2_O_2_; 16, plasmid + 50 μM Ru-but + 50 μM H_2_O_2_ + 1.4 mM DMSO; 17, plasmid + 50 μM Ru-but + 50 μM H_2_O_2_ + 40 mM M NaN_3_.; Table S2: IC_50_ (µM) values of the investigated complexes toward the selected cancer cell lines for 24 h.; Table S3: IC_50_ (µM) values of the investigated complexes toward the selected cancer cell lines for 48 h.
