# Supplementary material for: Two out of Three Musketeers Fight against Cancer: Synthesis, Physicochemical, and Biological Properties of Phosphino CuI, RuII, IrIII Complexes
Source: Pharmaceuticals (Basel). 2022 Jan 29;15(2):169. doi: 10.3390/ph15020169 (PMC8876511; doi:10.3390/ph15020169)
Supplement: Supplementary file 1 [file pharmaceuticals-15-00169-s001.zip › pharmaceuticals-1563645-supplementary.pdf]

Two out of Three Musketeers fight against cancer: synthesis, physicochemical and biological properties of phosphino Cu^{I}, Ru^{II}, Ir^{III} complexes

Urszula K. Komarnicka,[1]*Alessandro Niorettini,[2] Sandra Kozieł,[1] Barbara Pucelik,[3] Agata Barzowska,[3] Daria Wojtala,[1] Aleksandra Ziółkowska,[1] Monika Lesiów,[1] Agnieszka Kyzioł,[4] Stefano Caramori,[2] Marina Porchia,[5] Alina Bieńko[1]

[1] Faculty of Chemistry, University of Wroclaw, Joliot-Curie 14, 50-383 Wroclaw, Poland; monika.lesiow@chem.uni.wroc.pl; daria.wojtala@wp.pl; alina.bienko@chem.uni.wroc.pl; daria.wojtala@chem.uni.wroc.pl
[2] Department of Chemical, Pharmaceutical, and Agricultural Sciences, University of Ferrara, Via L. Borsari 46, 44121 Ferrara, Italy; cte@unife.it; alessandro.niorettini@unife.it
[3] Małopolska Centre of Biotechnology, Jagiellonian University, Gronostajowa 7A, 30-387, Kraków, Poland, barbara.pucelik@gmail.com, agata.barzowska@doctoral.uj.edu.pl
[4] Faculty of Chemistry, Jagiellonian University in Krakow, Gronostajowa 2, 30-387 Krakow, Poland, kyziol@chemia.uj.edu.pl
[5] ICMATE-CNR, Corso Stati Uniti, 4, 35127 Padova, Italy

*Correspondence: U.K.K.: urszula.komarnicka@chem.uni.wroc.pl

Citation: Komarnicka, U.K.; Niorettini, A.; Kozieł, S.; Pucelik,B.; Barzowska, A.; Wojtala, D.; Ziółkowska, A.; Lesiów, M.; Kyzioł, A.; Caramori, C.; et al. *Two out of Three Fight Against Cancer*: Synthesis, Physicochemical and Biological Properties of Phosphino Cu^{I}, Ru^{II}, Ir^{III} complexes. *Pharmaceuticals* **2022**, *14*, x. https://doi.org/10.3390/ph15020169

Academic Editor: Maria Emília de Sousa

Received: 3 January 2022
Accepted: 25 January 2022
Published: 29 January 2022

Publisher's Note: MDPI stays neutral with regard to jurisdictional claims in published maps and institutional affiliations.

Copyright:© 2021by the authors. Submitted for possible open access publication under the terms and conditions of the Creative Commons Attribution (CC BY) license (https://creativecommons.org/licenses/by/4.0/).

Table S1 Cumulative NMR data for ligands and iridium-complexes.

	**1**	**2**	**[Ir(η^5-Cp*)Cl$_2$]$_2$**	**[Ru(*p*-cymene)Cl$_2$]$_2$**	**1a**	**1b**	**1c**	**2a**	**2b**	**2c**
**$^{31}P\{^1H\}$**	**-27.68**	**-26.72**			**-1.78**	**24.32**	**-14.49**	**-2.01**	**23.92**	**-13.07**
**H^{Ph}**		7.25-7.64			7.24-7.61 m	7.31-8.02 m	7.28-7.49	7.30-8.02	7.41-8.04	7.25 – 7.32
**H^1**	3.45 s	3.48 s			4.01 s*	3.89 s*	3.49 s*	4.02 s	4.01 s	3.62 s
**H^2**	2.79 q	2.64 d			2.25 s*	2.19 s*	2.20 s*	2.09 s	1.98 s*	2.08 s
**H^3**	1.01 t	1.34-1.50 m			0.72 s*	1.31 s*	0.89 s*	0.72 – 0.98 m	0.70-0.89 m	0.82 -1.24 m
**H^4**		1.34-1.50 m						0.72 – 0.98 m	0.70-0.89 m	0.82 -1.24 m
**H^5**		0.89 t						0.71 t	0.68 t	0.76 t
**$H^{acetonitrille}$**							2.03 s			2.02 s
**$H^{Cp*(CH3)}$**			0.96 s		1.47 s*			1.37 s		
**$H^{a,b}$**				1.29 d (6.9)		0.84 d			1.08 d	
**H^c**				2.93 spt (7.0)		2.97 (6.80) spt			2.51 spt	
**$H^{e,f,h,i}$**				5.42 dd (19.2; 6.0)		5.37 (36.0; 7.0) dd			5.15 dd	
**H^j**				2.16 s		1.74 s			1.75 s	

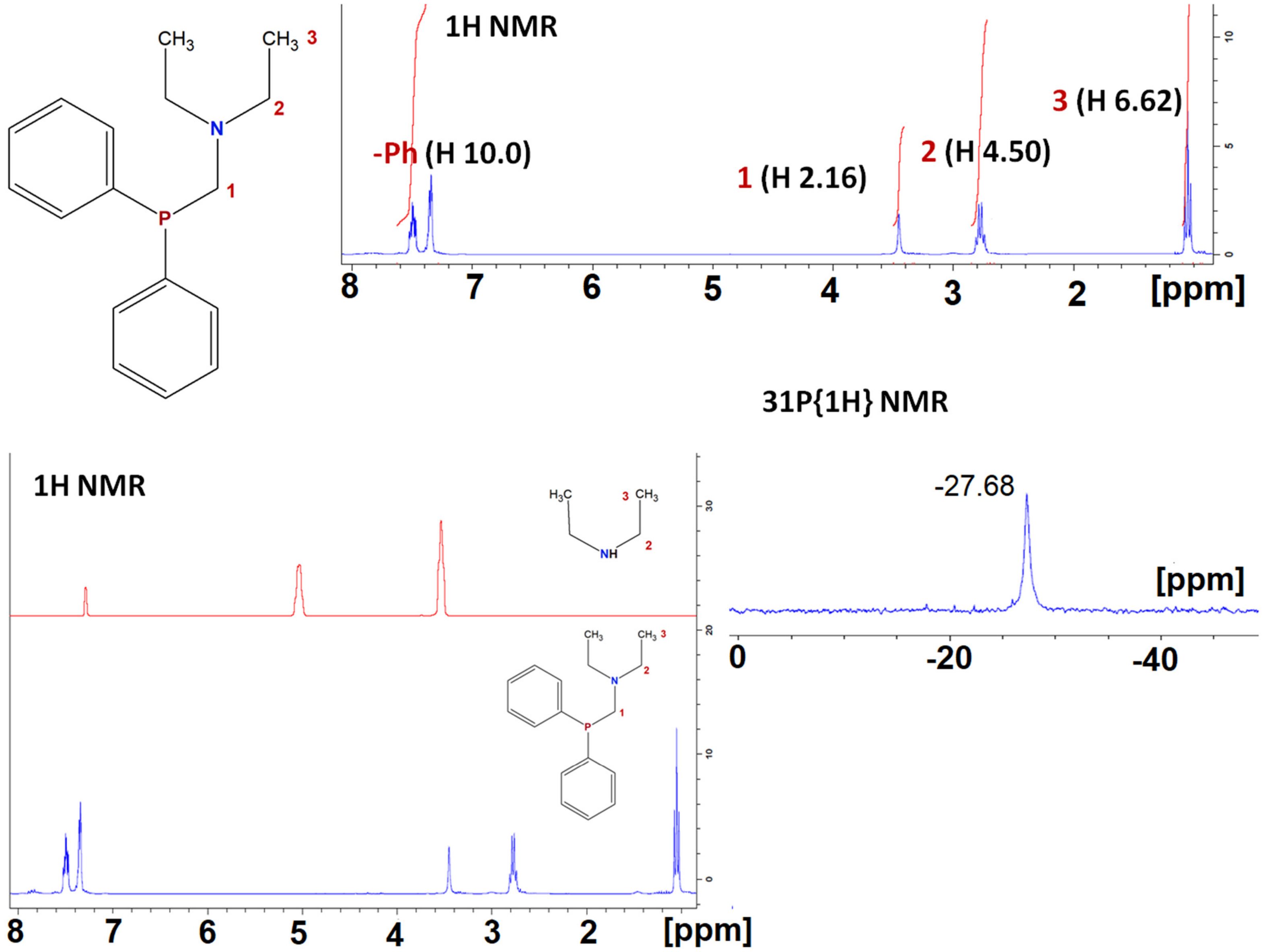

Figure S1 ^{1}H and ^{31}P{^{1}H} NMR spectra for **1** (298 K, $CHCl_3$-d)

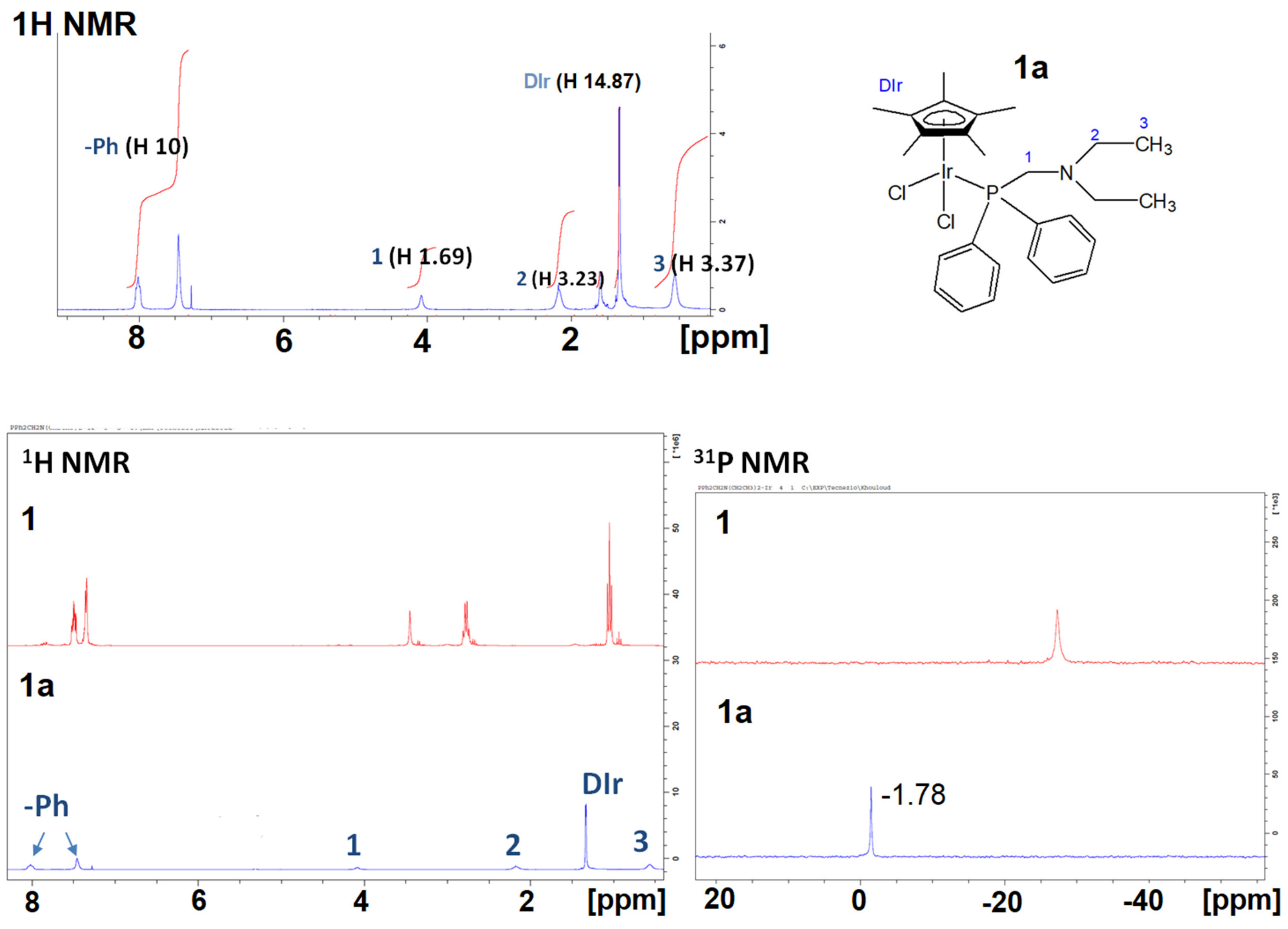

Figure S2 ^{1}H and ^{31}P{^{1}H} NMR spectra for **1a** (298 K, $CHCl_3$-d)

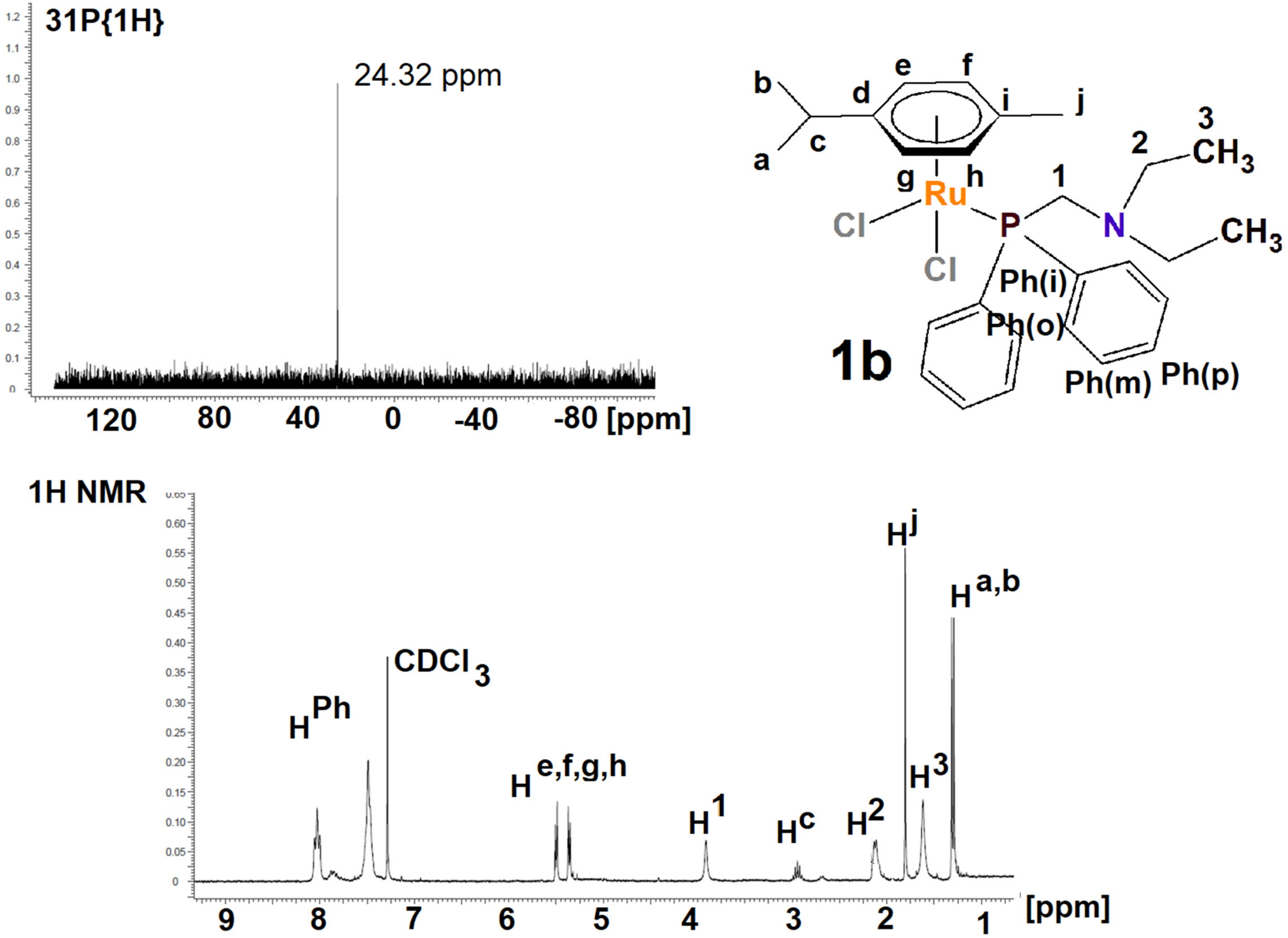

Figure S3 ^{1}H and $^{31}P\{^{1}H\}$ NMR spectra for **1b** (298 K, $CHCl_3$-d)

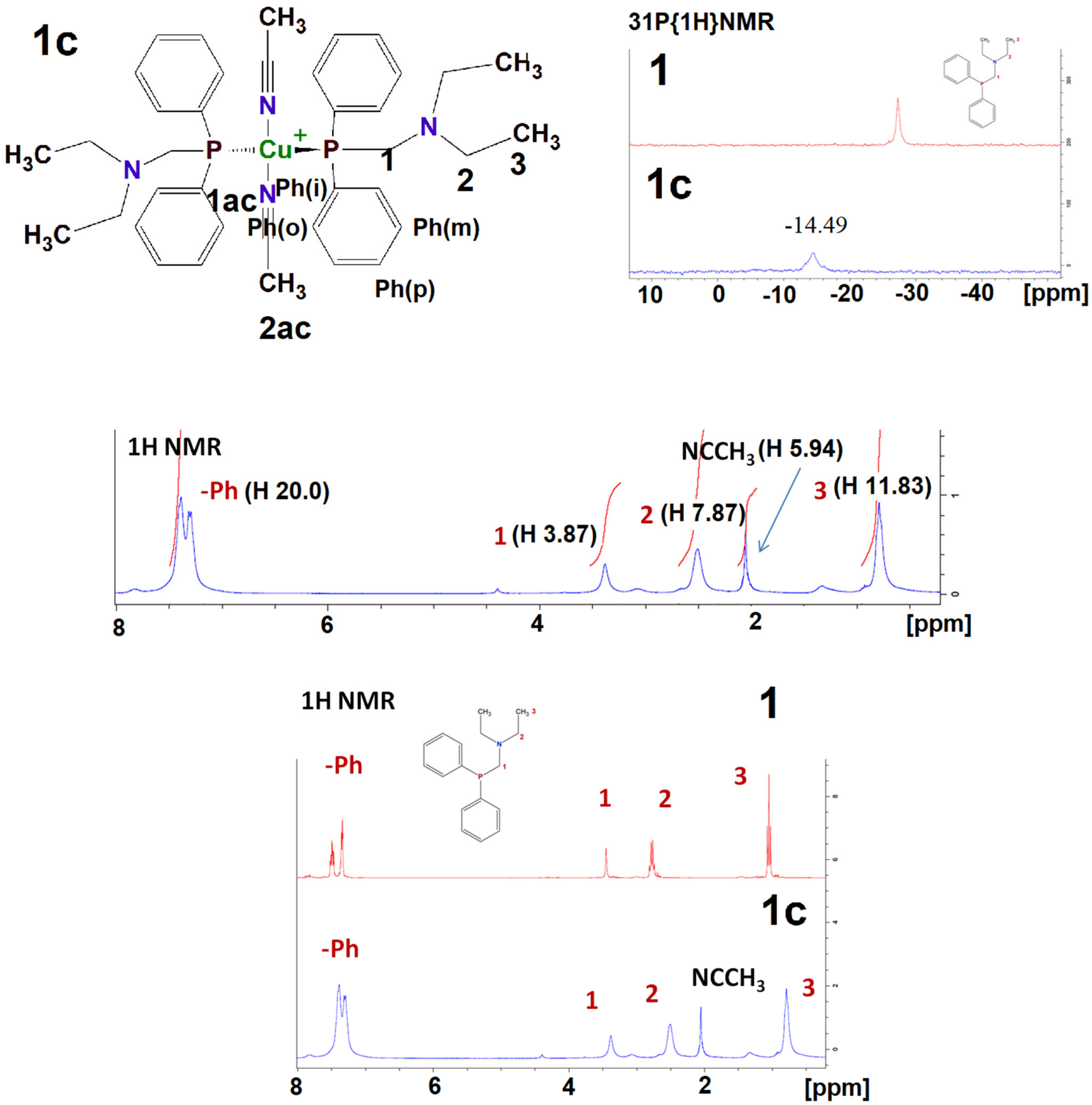

Figure S4 ^{1}H and ^{31}P{^{1}H} NMR spectra for **1c** (298 K, $CHCl_3$-d)

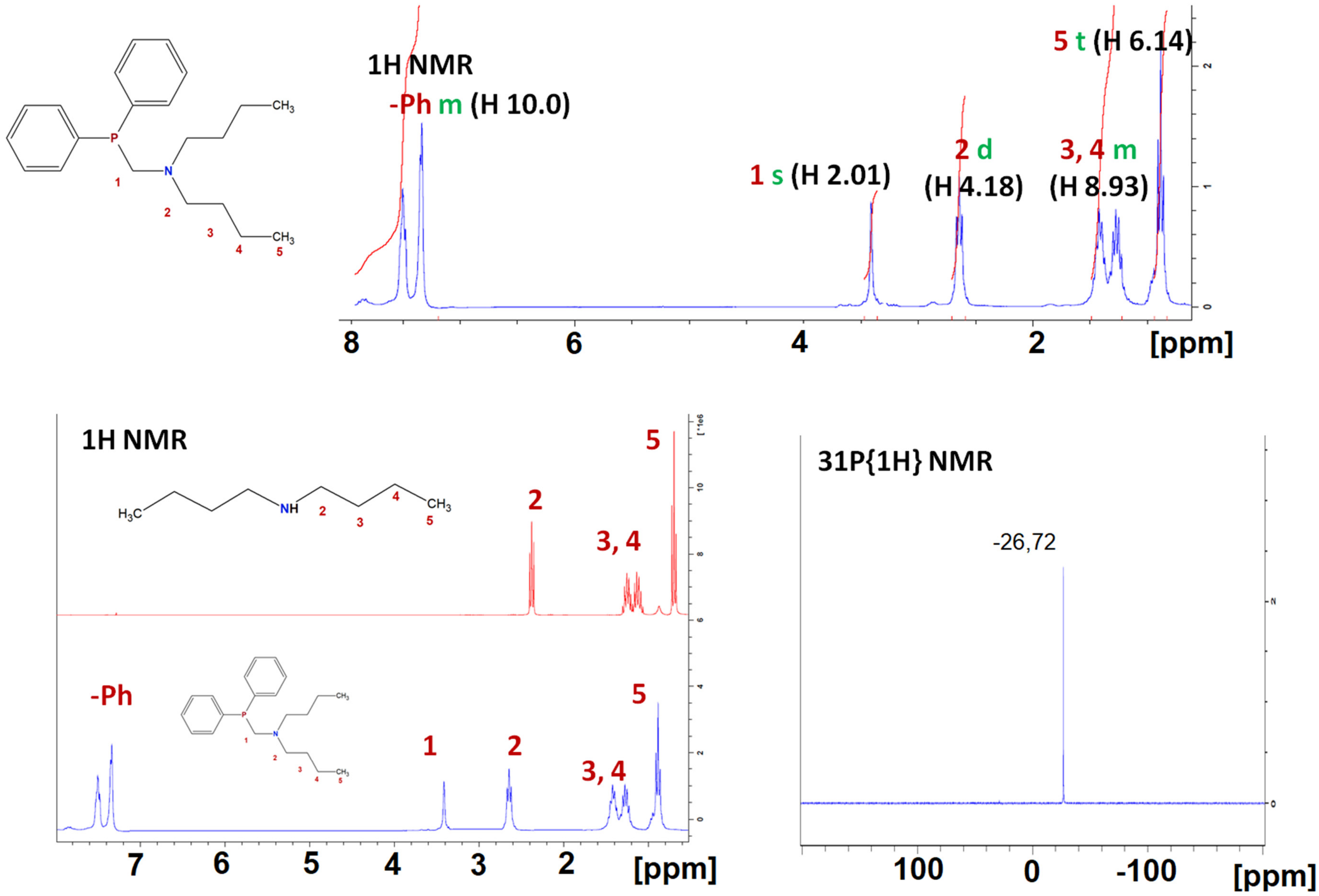

Figure S5 ^{1}H and $^{31}P\{^{1}H\}$ NMR spectra for **2** (298 K, $CHCl_3$-d)

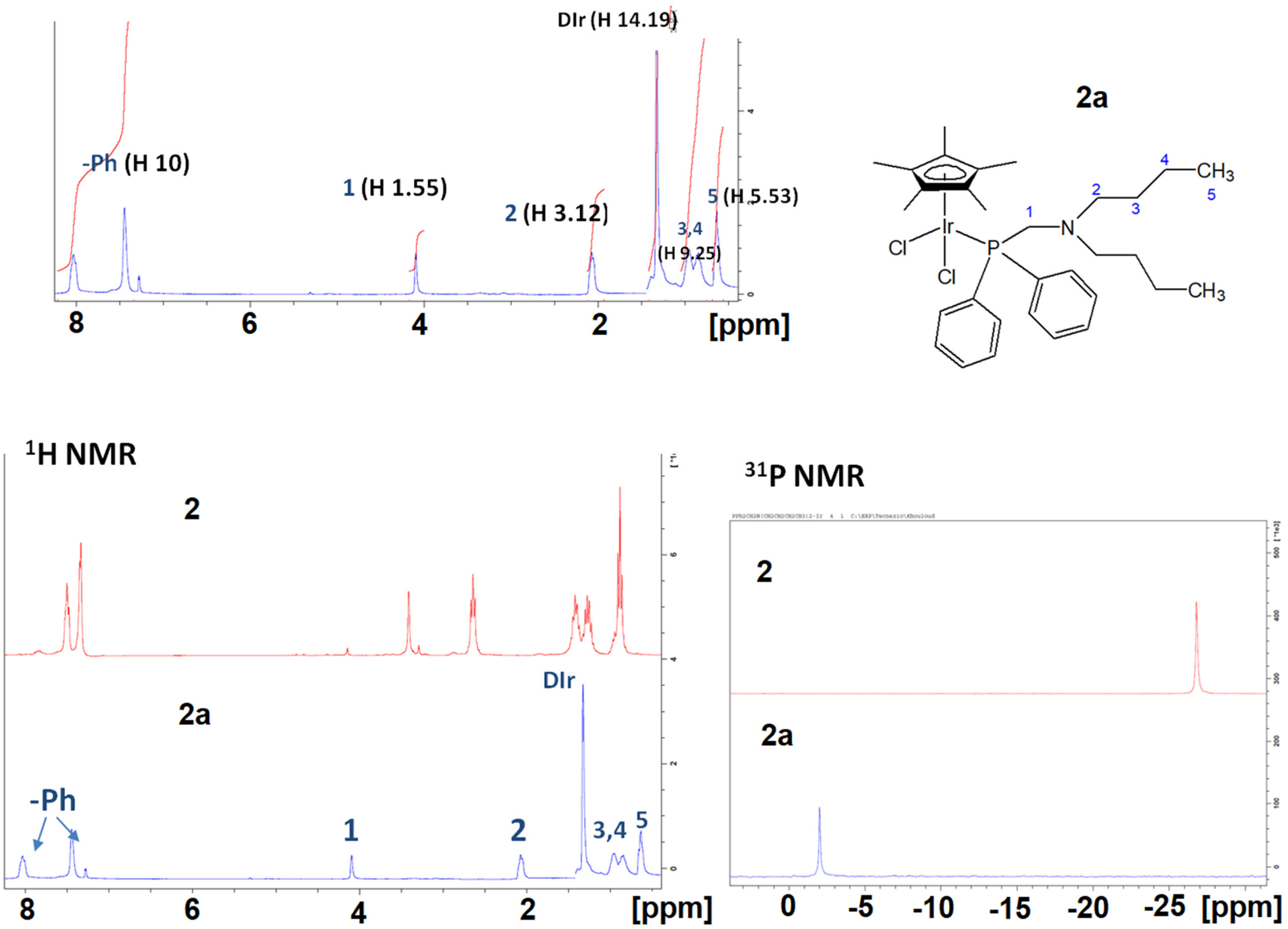

Figure S6 ^{1}H and $^{31}P\{^{1}H\}$ NMR spectra for **2a** (298 K, $CHCl_3$-d)

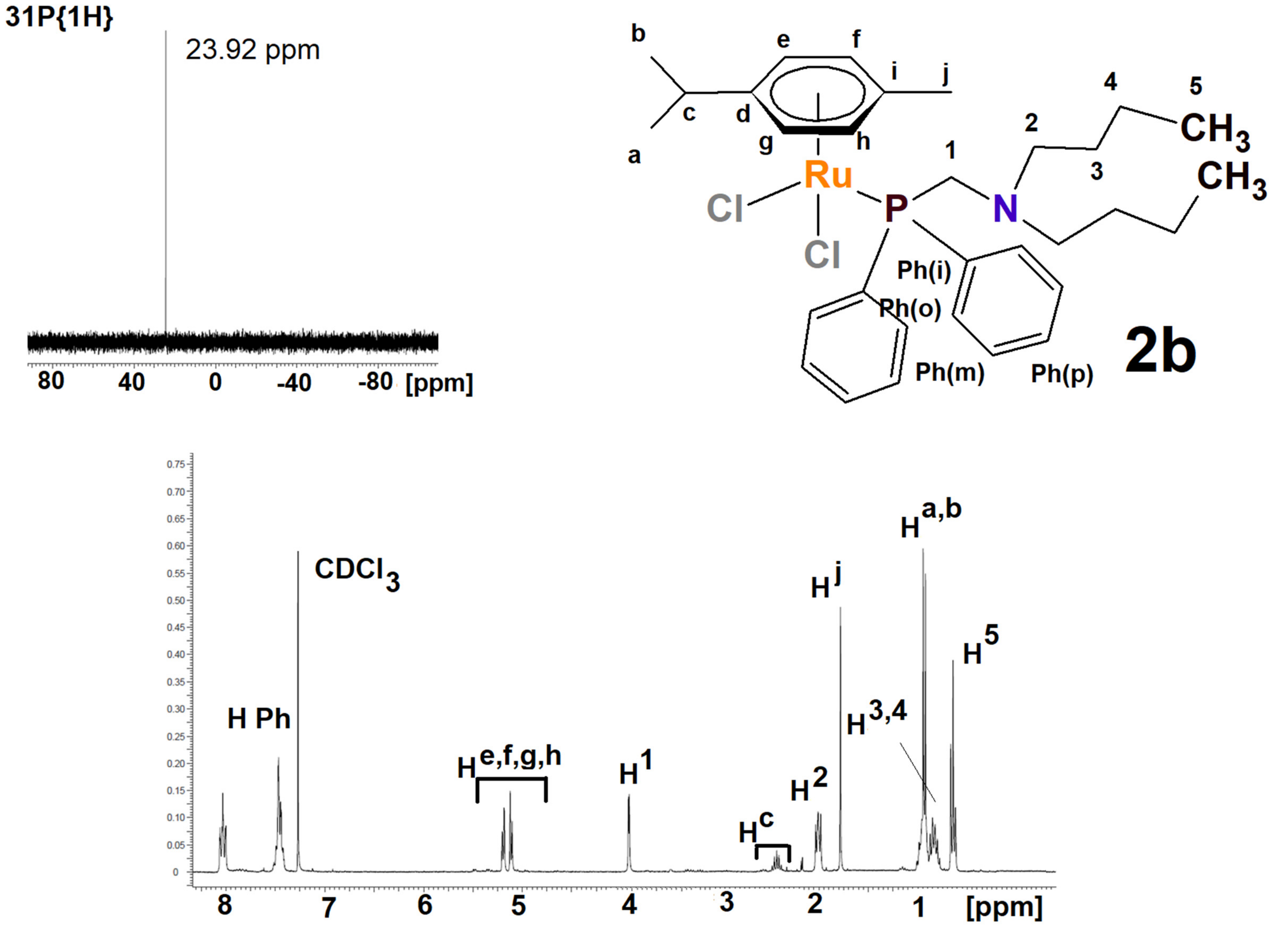

Figure S8 ^{1}H and ^{31}P{^{1}H} NMR spectra for **2b** (298 K, $CHCl_3$-d)

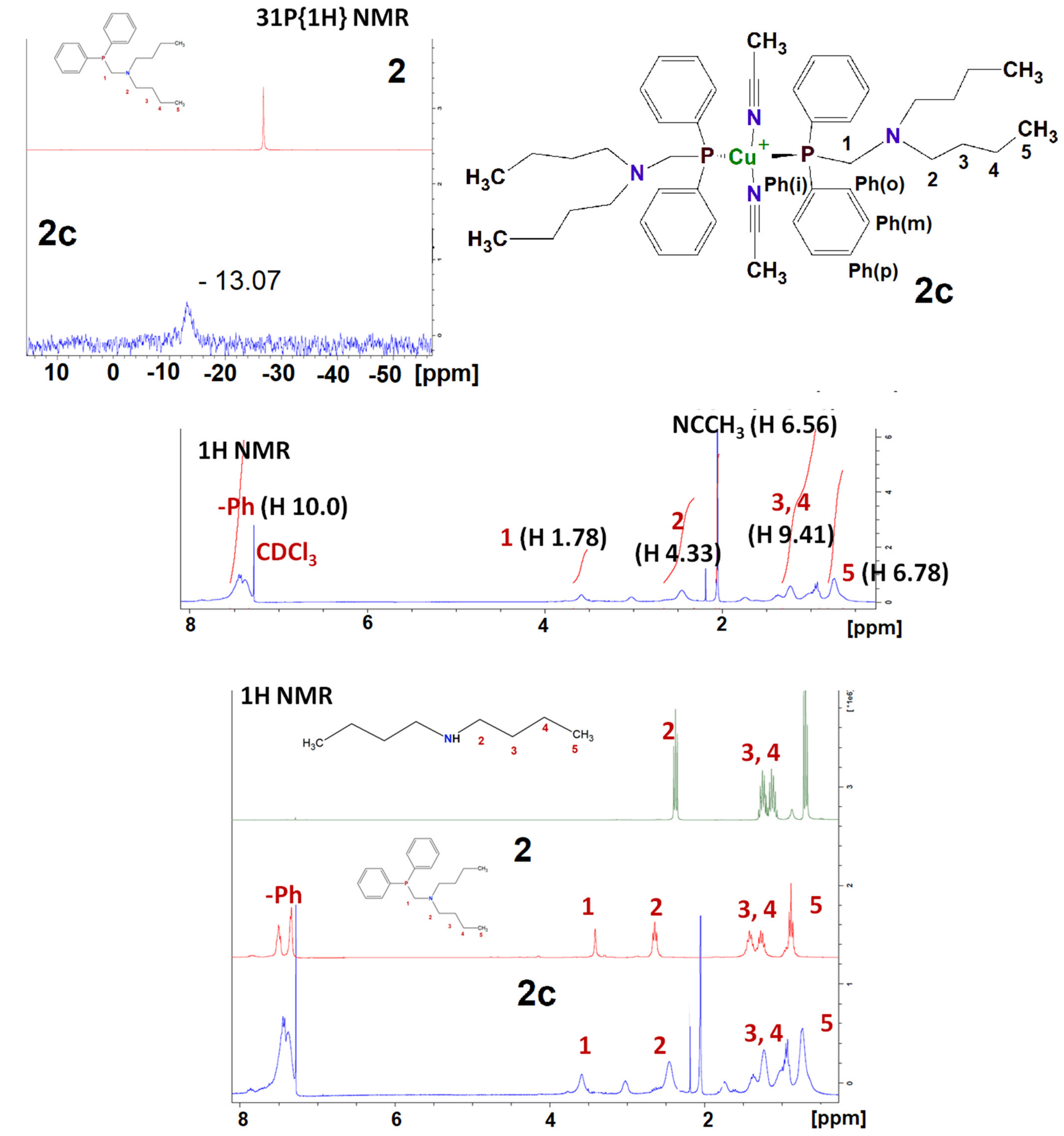

Figure S9 ^{1}H and $^{31}P\{^{1}H\}$ NMR spectra for **2c** (298 K, $CHCl_3$-d)

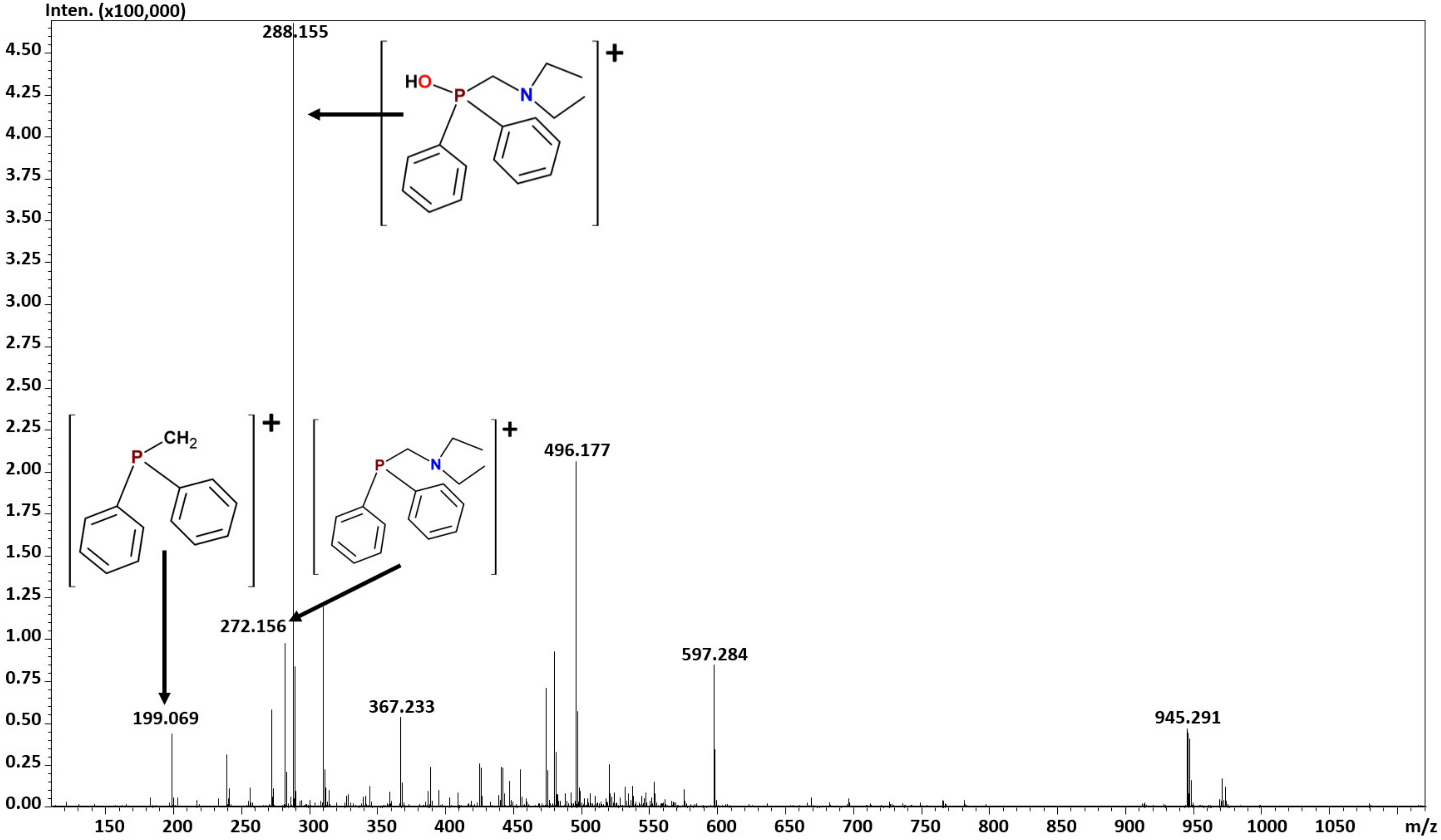

Figure S10 Full ESI(+)MS spectrum of **1**.

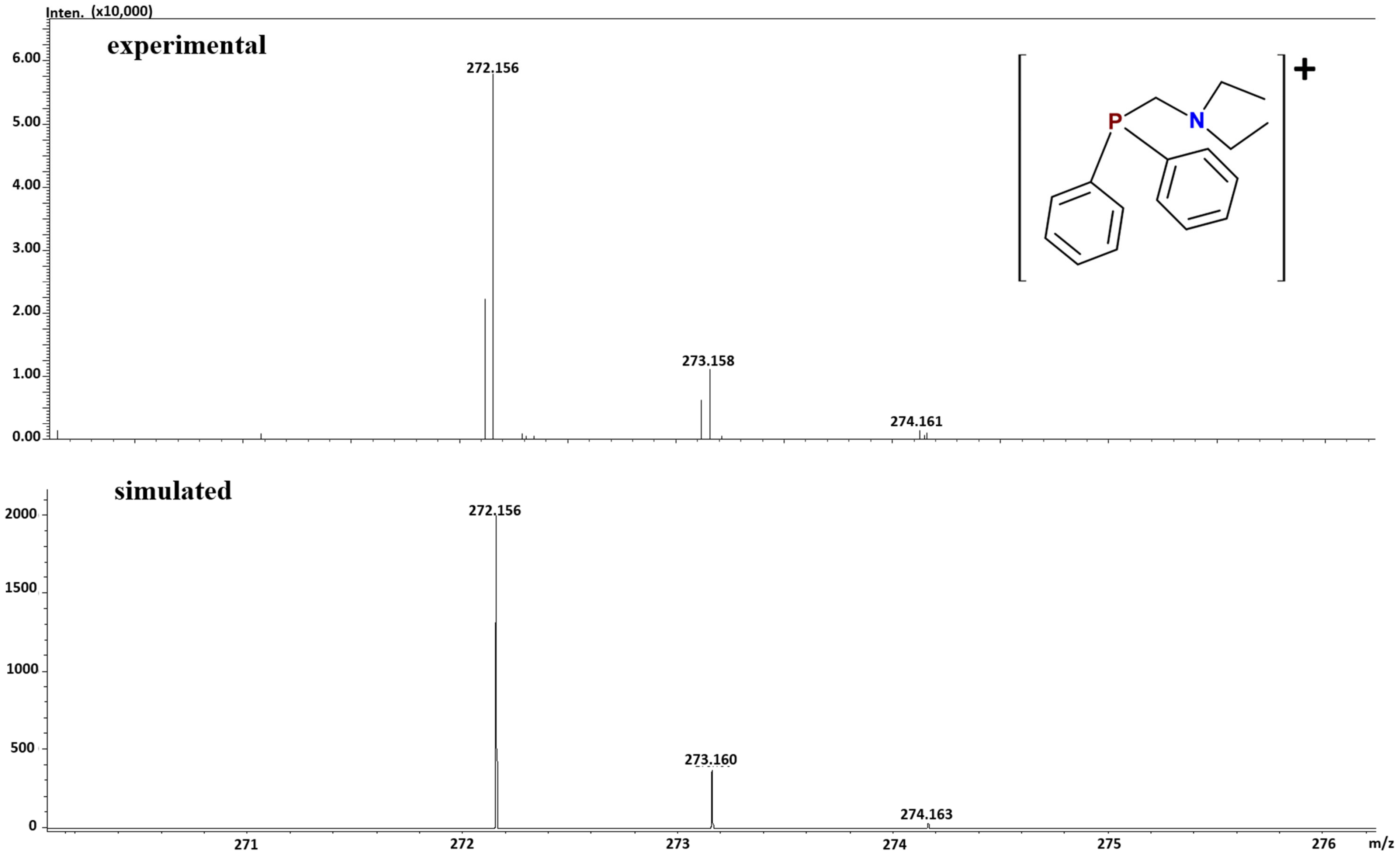

Figure S11 Experimental and simulated ESI(+)MS spectrum of **1**.

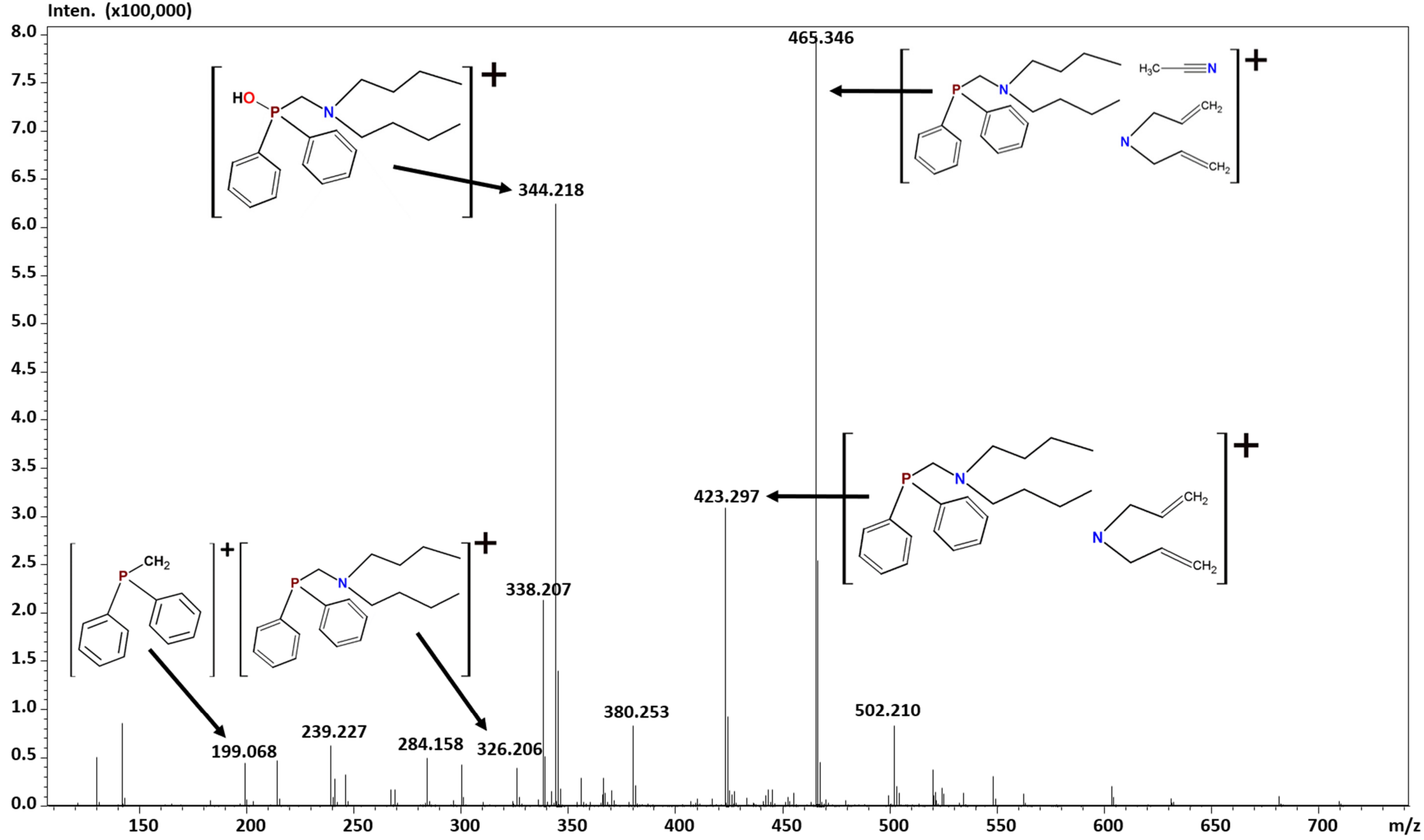

Figure S12 Full ESI(+)MS spectrum of **2**.

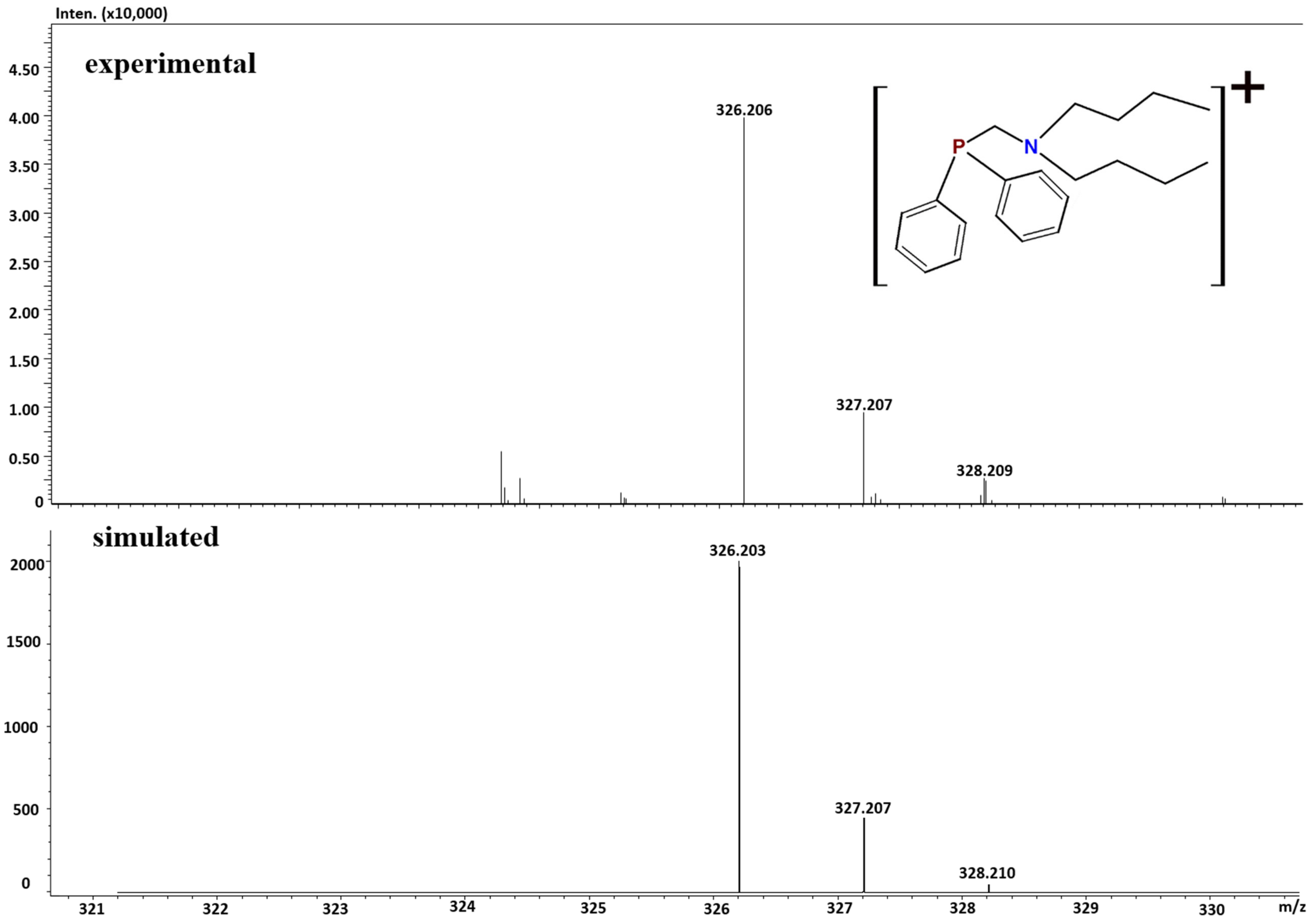

Figure S13 Experimental and simulated ESI(+)MS spectrum of **2**.

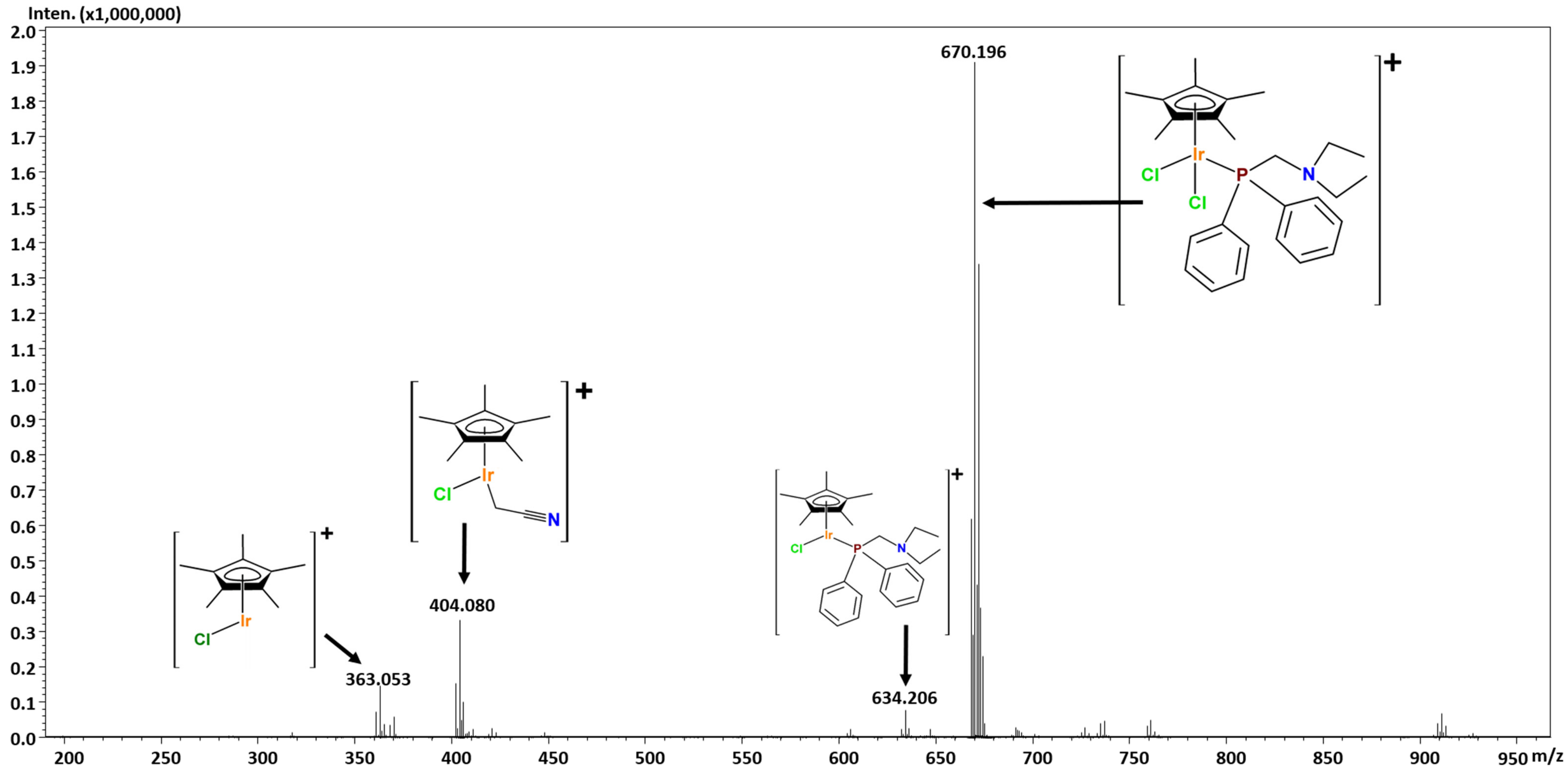

Figure S14 Full ESI(+)MS spectrum of **1a**.

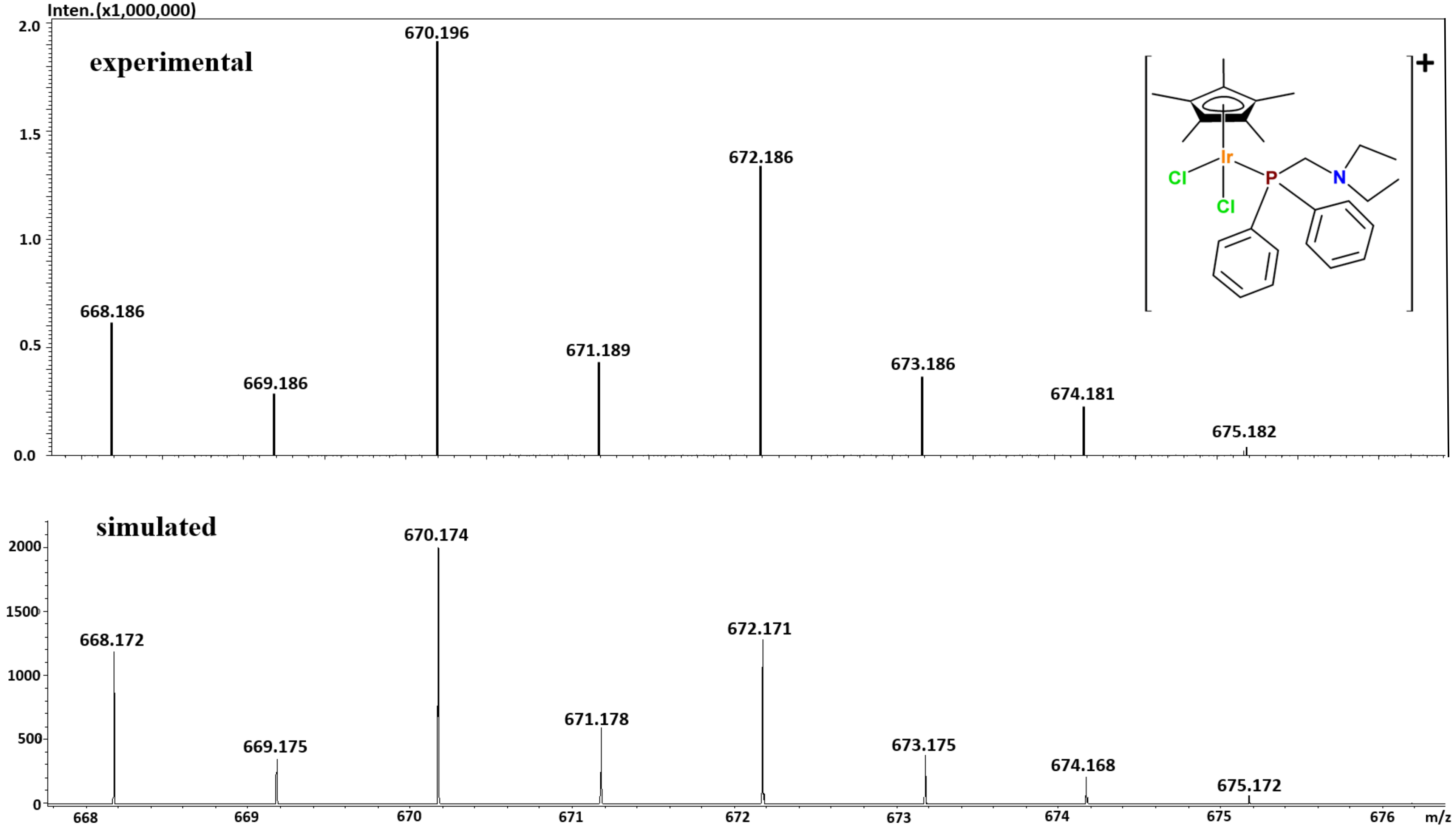

Figure S15 Experimental and simulated ESI(+)MS spectrum of **1a**.

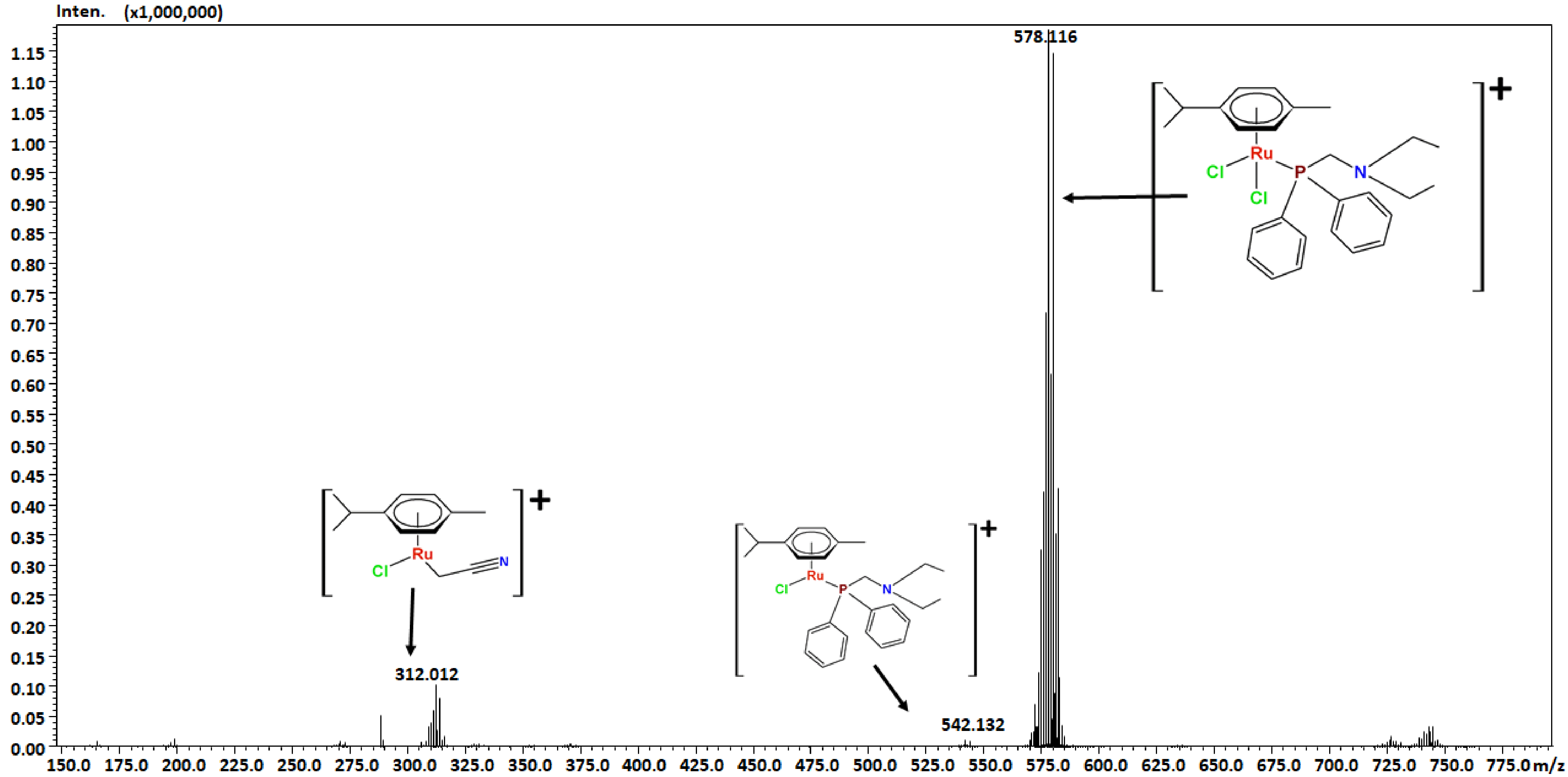

Figure S16 Full ESI(+)MS spectrum of **1b**.

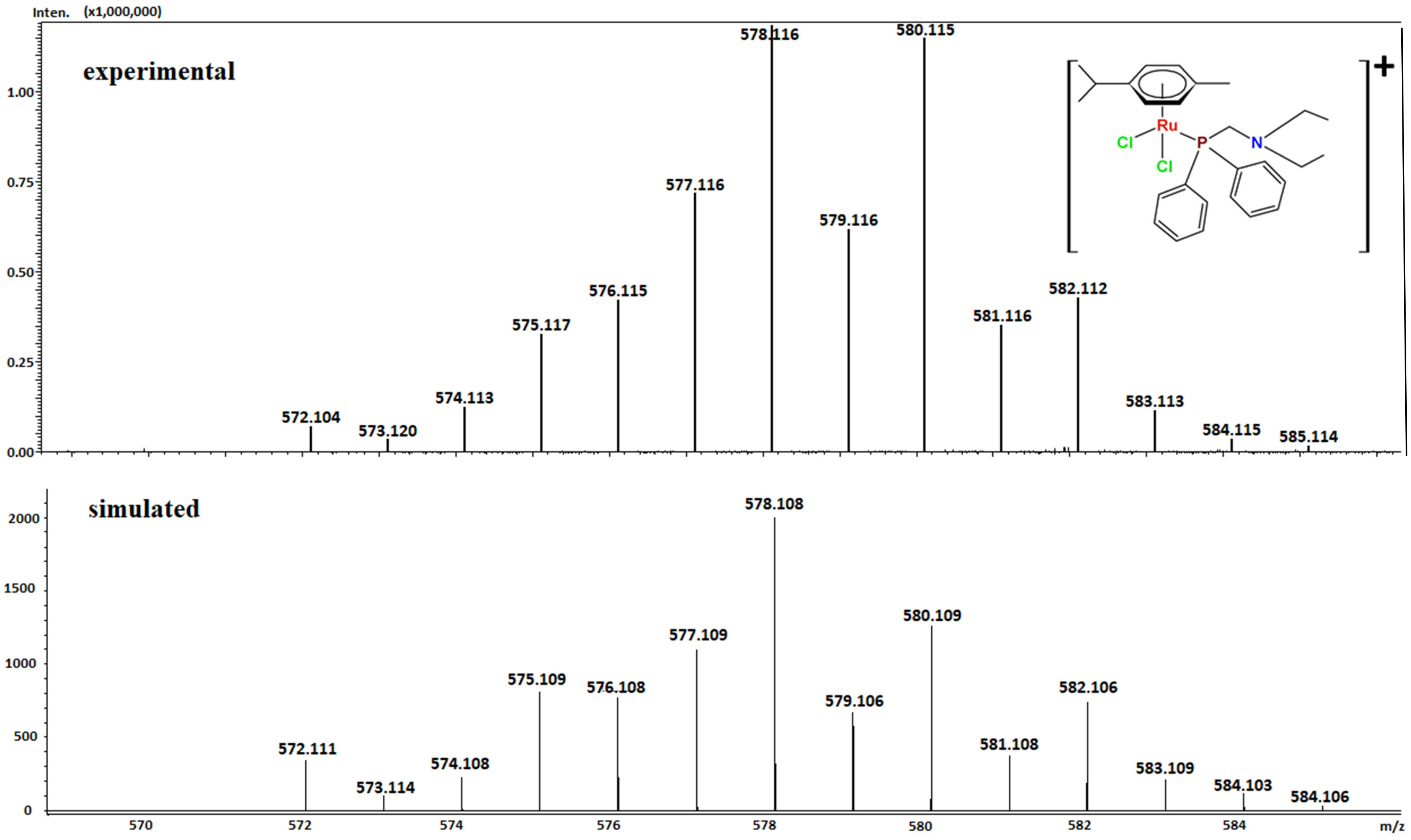

Figure S17 Experimental and simulated ESI(+)MS spectrum of **1b**.

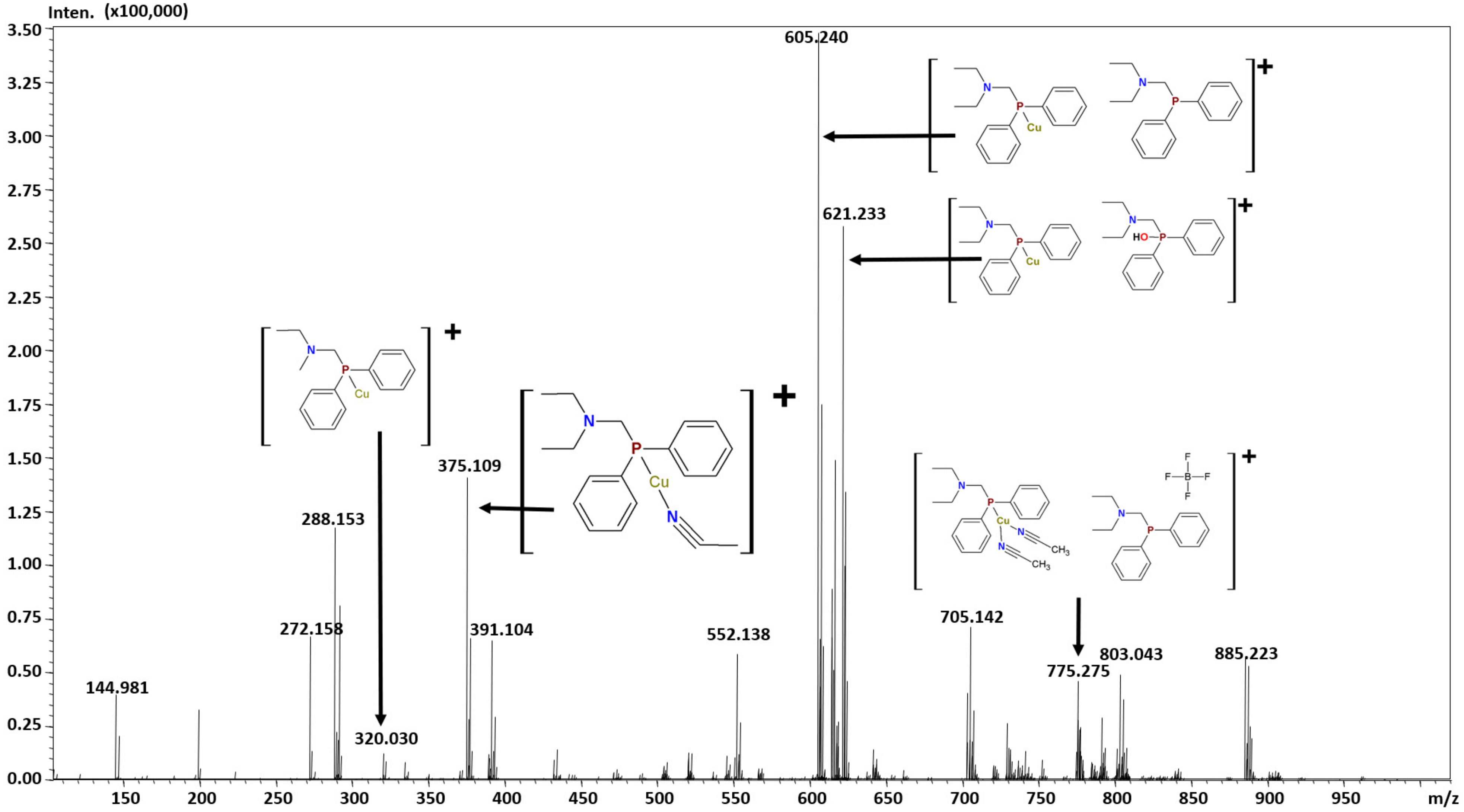

Figure S18 Full ESI(+)MS spectrum of **1c**.

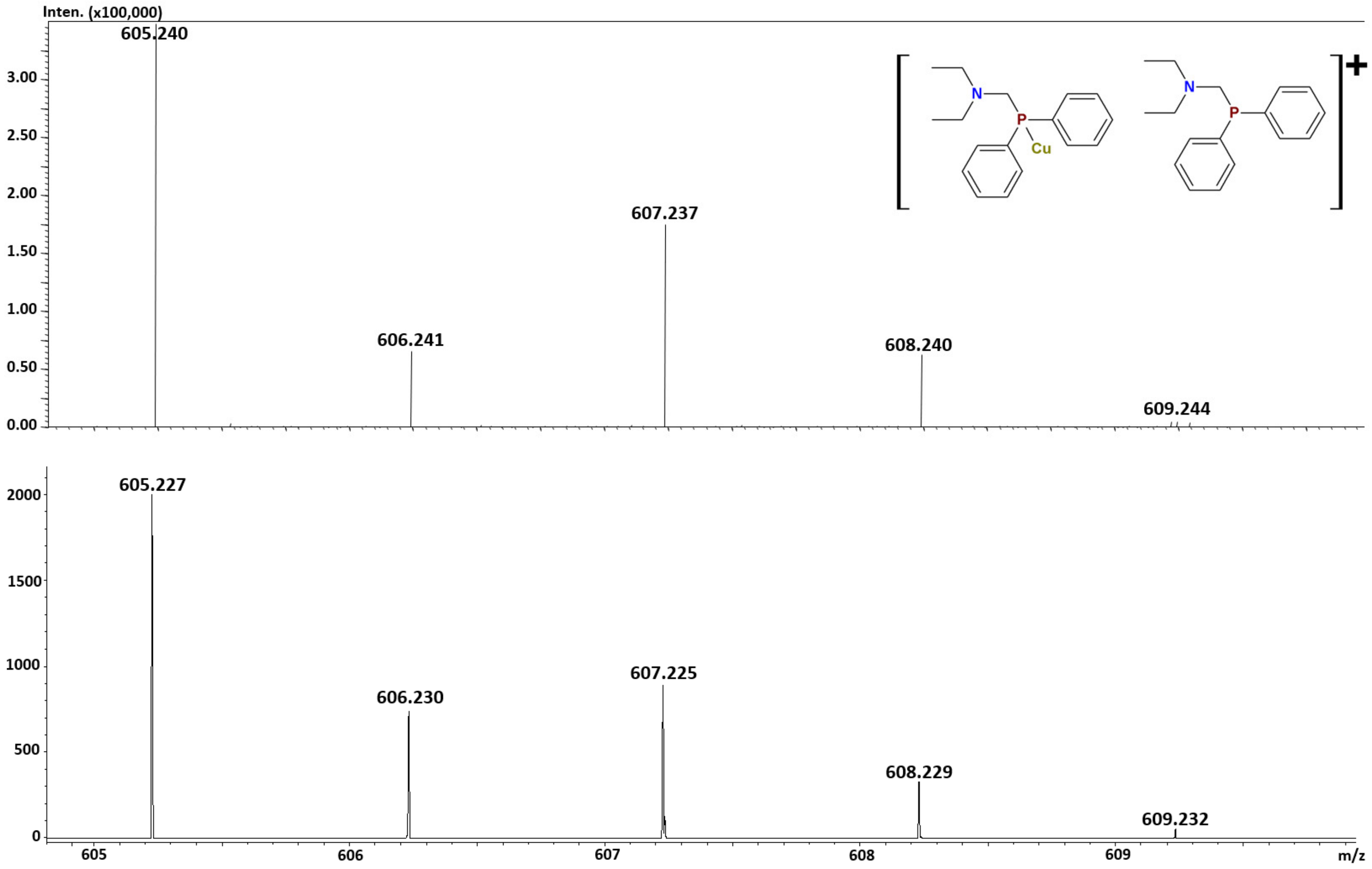

Figure S19 Experimental and simulated ESI(+)MS spectrum of **1b**.

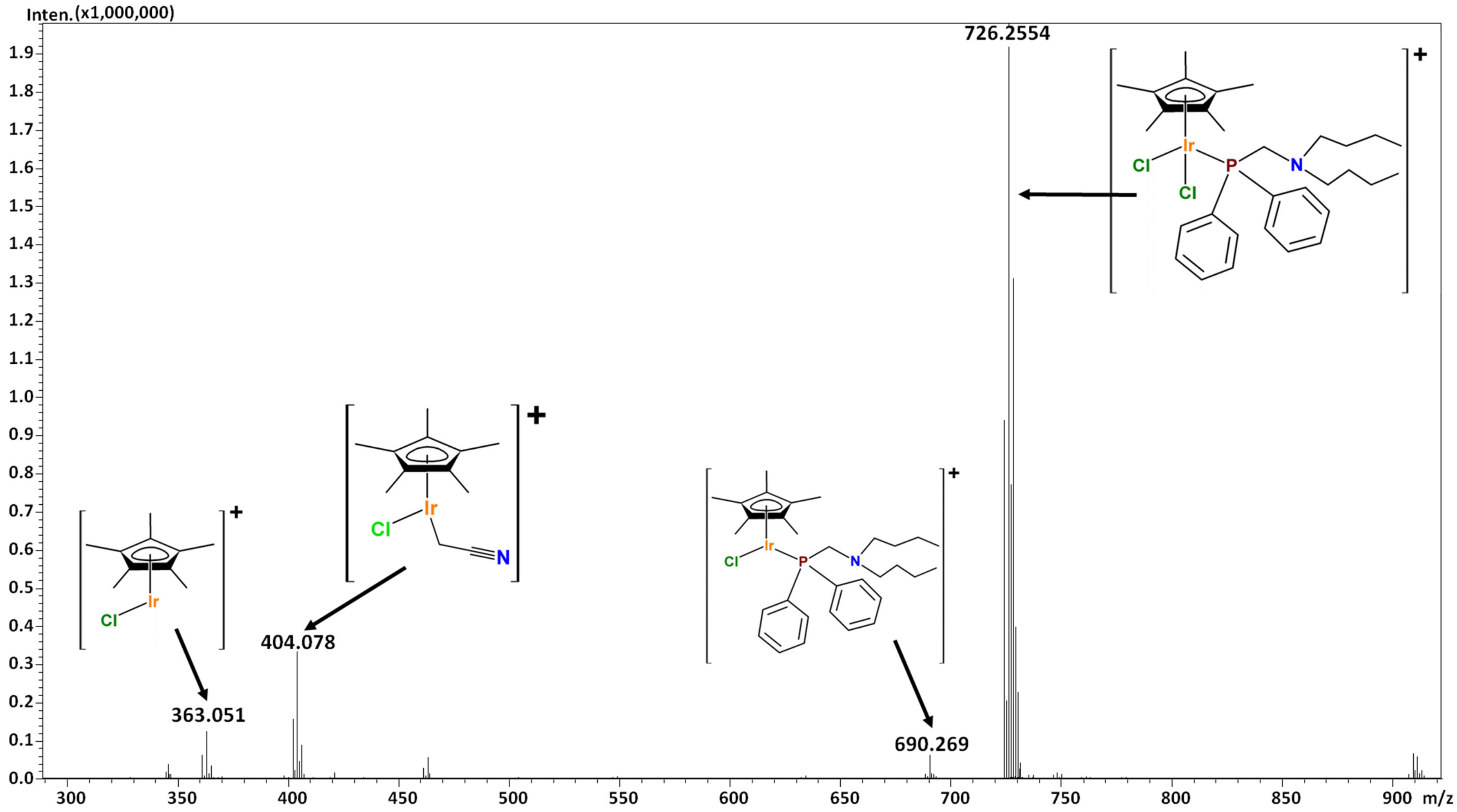

Figure S20Full ESI(+)MS spectrum of **2a**.

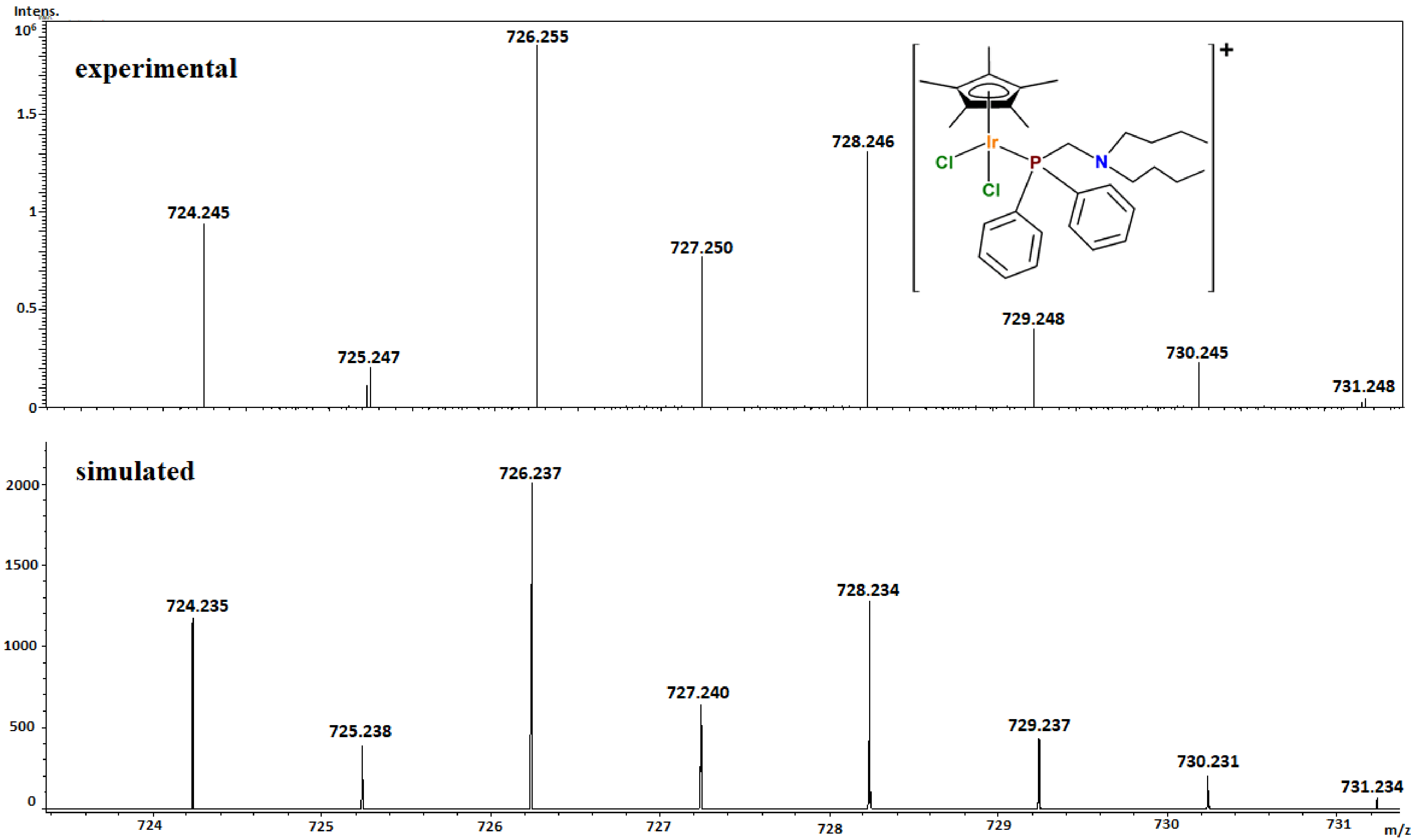

Figure S19 Experimental and simulated ESI(+)MS spectrum of **2a**.

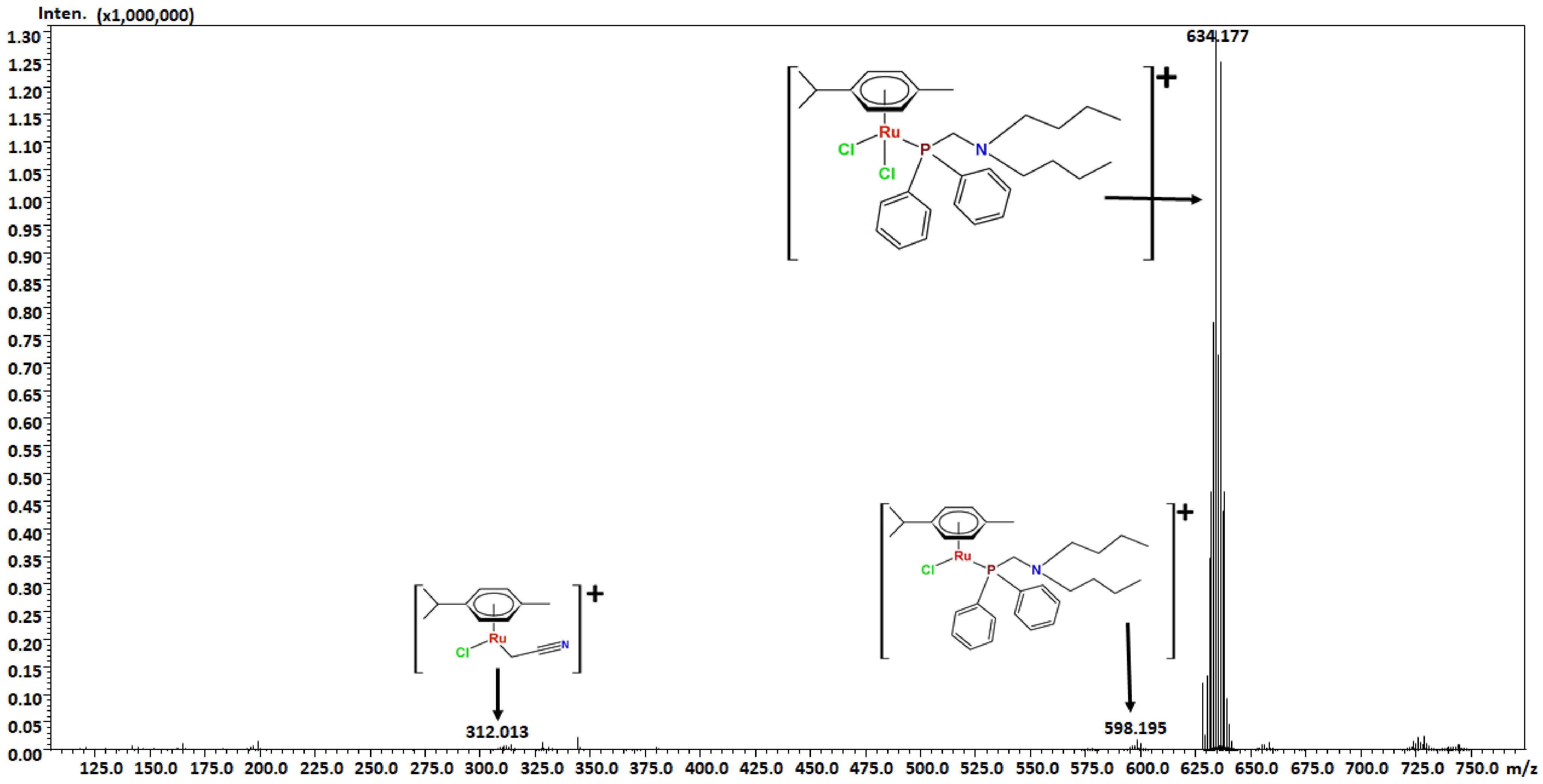

Figure S20 Full ESI(+)MS spectrum of **2b**.

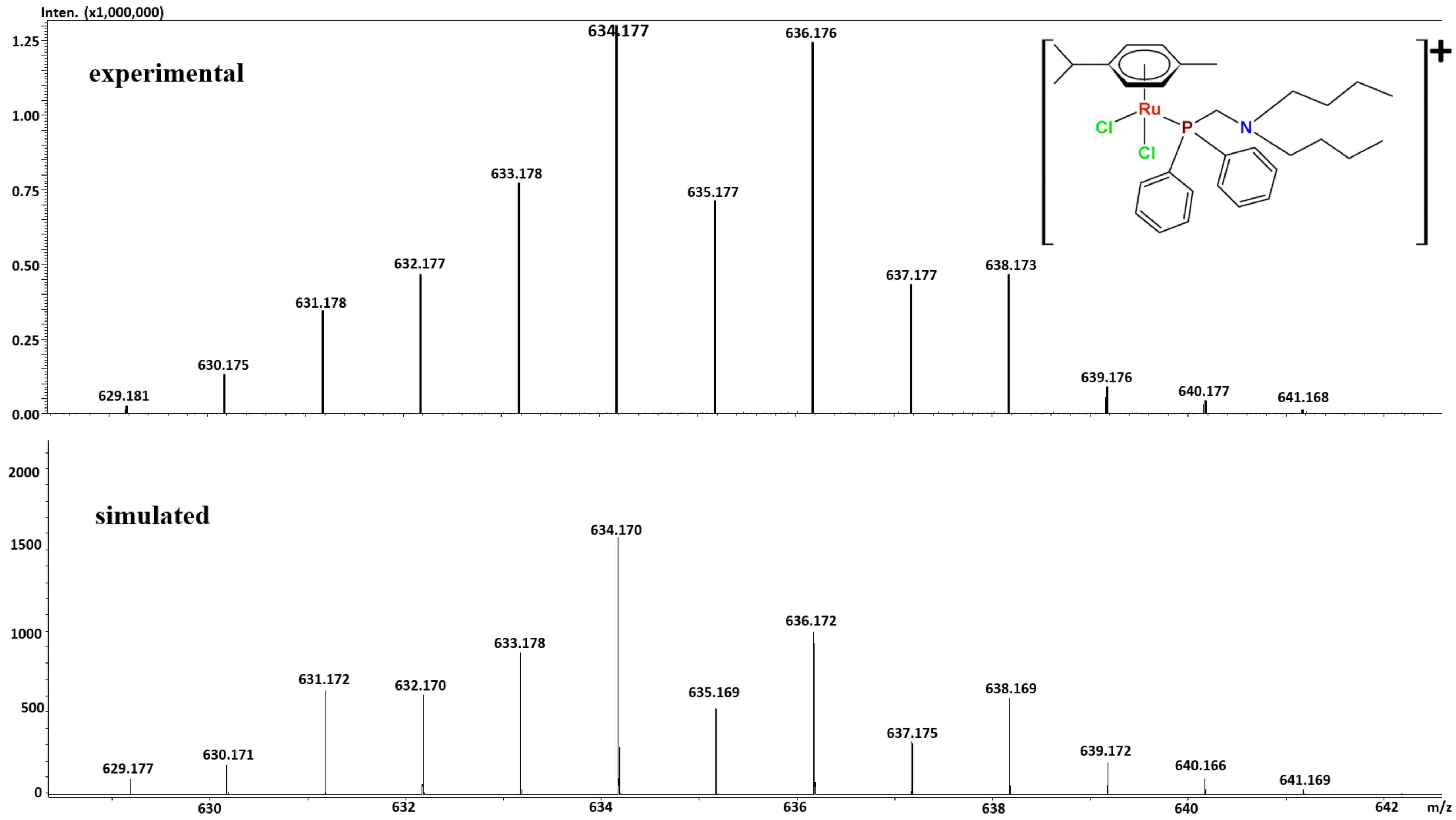

Figure S21 Experimental and simulated ESI(+)MS spectrum of **2b**.

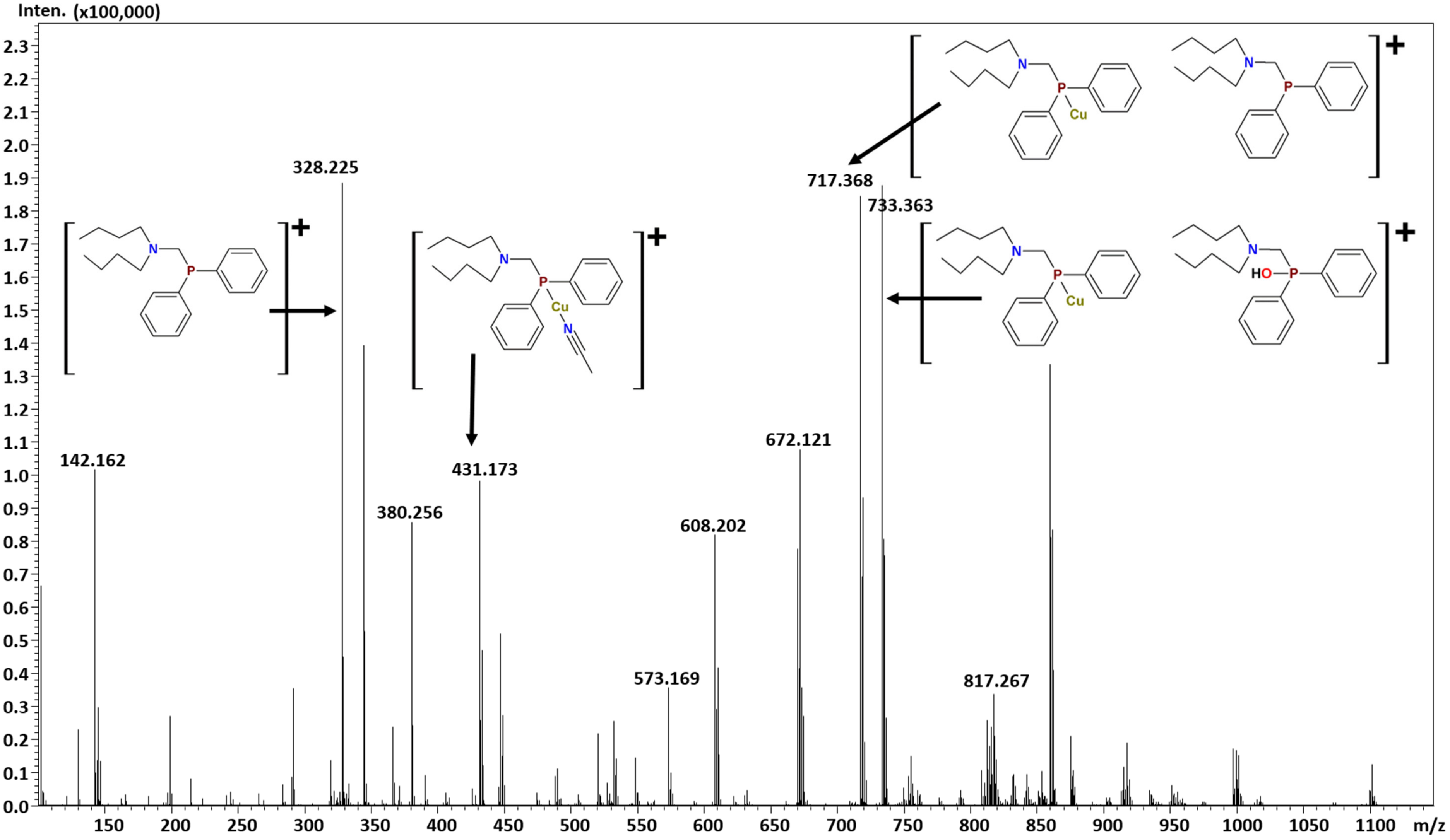

Figure S22 Full ESI(+)MS spectrum of **2c**.

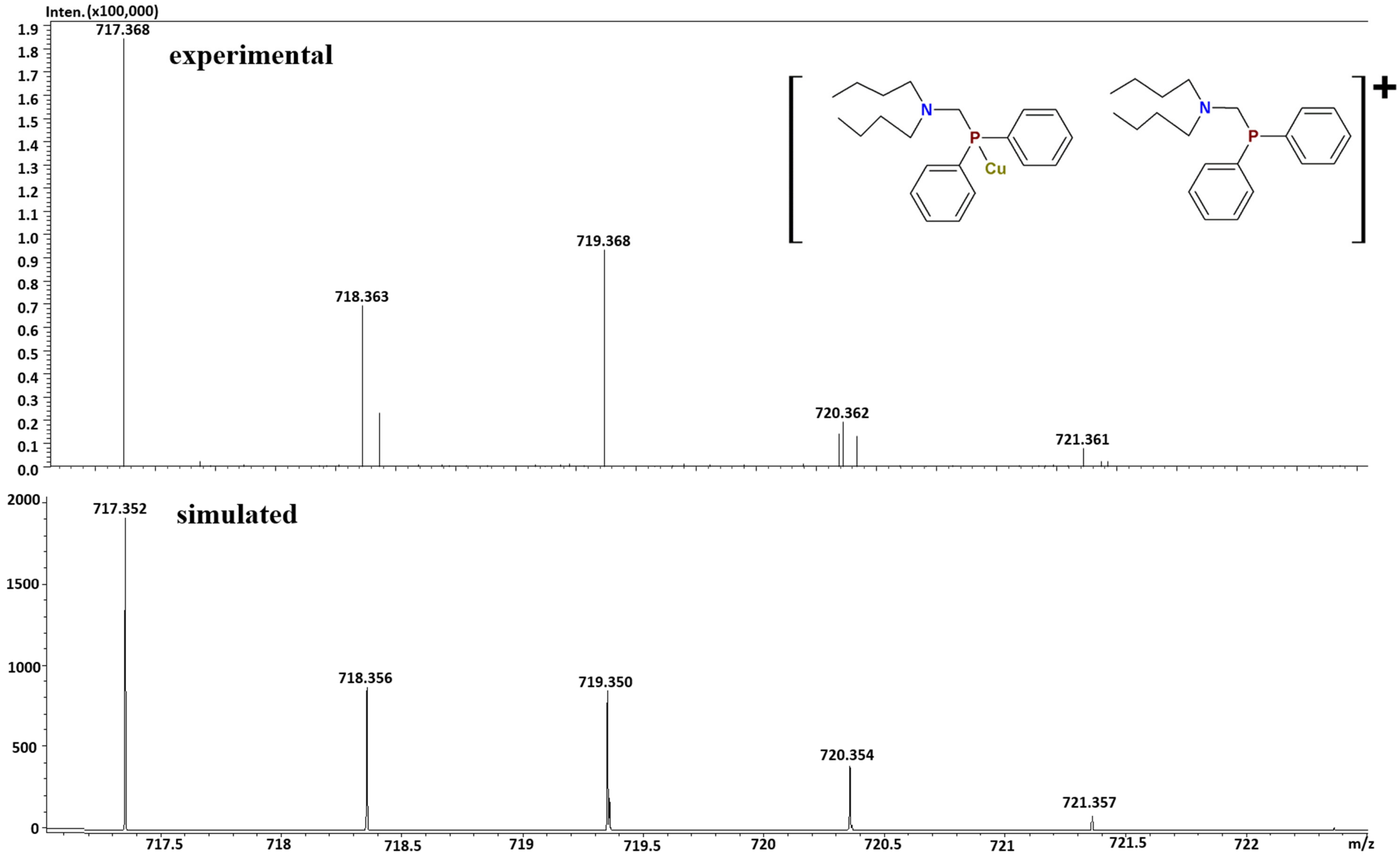

Figure S23 Experimental and simulated ESI(+)MS spectrum of **2c**.

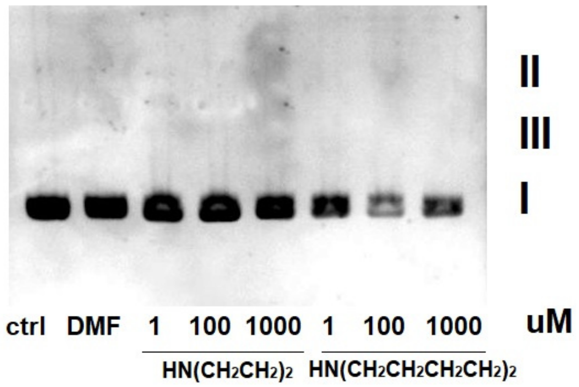

Figure S24 Agarose gel electrophoresis of pBR322 plasmid cleavage by diethyloamine and dibuthyloamine.

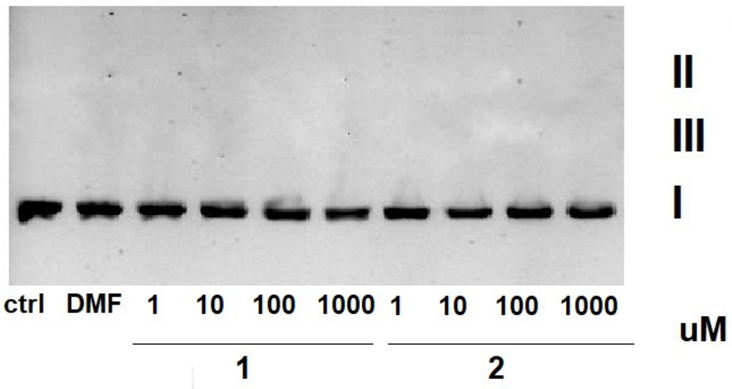

Figure S25 Agarose gel electrophoresis of pBR322 plasmid cleavage by phosphines

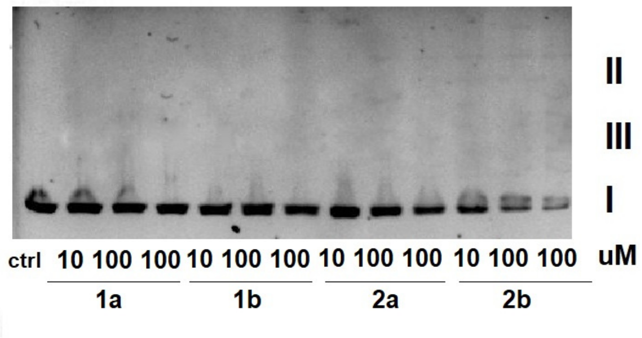

Figure S26 Agarose gel electrophoresis of pBR322 plasmid cleavage by Cu(I), Ru(II) and Ir(III) complexes.

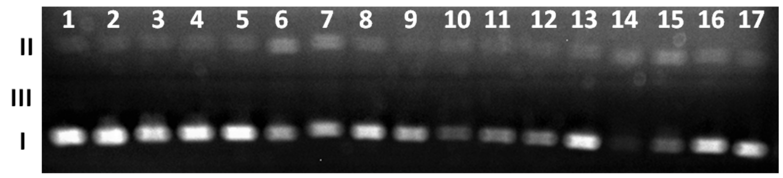

Figure S27 Agarose gel electrophoresis of pBR322 plasmid cleavage by Ir-ethyl, Ru-ethyl, Ir-but and Ru-but complexes. Lanes: 1, plasmid–control; 2, plasmid + 50 μM Ir-ethyl; 3, plasmid + 50 μM Ir-ethyl + 50 μM H_2O_2; 4, plasmid + 50 μM Ir-ethyl + 50 μM H_2O_2 + 1.4 mM DMSO; 5, plasmid + 50 μM Ir-ethyl + 50 μM H_2O_2 + 40 mM M NaN_3; 6, plasmid + 50 μM Ru-ethyl; 7, plasmid + 50 μM Ru-ethyl + 50 μM H_2O_2; 8, plasmid + 50 μM Ru-ethyl + 50 μM H_2O_2 + 1.4 mM DMSO; 9, plasmid + 50 μM Ru-ethyl + 50 μM H_2O_2 + 40 mM M NaN_3; 10, plasmid + 50 μM Ir-but; 11, plasmid + 50 μM Ir-but + 50 μM H_2O_2; 12, plasmid + 50 μM Ir-but + 50 μM H_2O_2 + 1.4 mM DMSO; 13, plasmid + 50 μM Ir-but + 50 μM H_2O_2 + 40 mM NaN_3; 14, plasmid + 50 μM Ru-but; 15, plasmid + 50 μM Ru-but + 50 μM H_2O_2; 16, plasmid + 50 μM Ru-but + 50 μM H_2O_2 + 1.4 mM DMSO; 17, plasmid + 50 μM Ru-but + 50 μM H_2O_2 + 40 mM M NaN_3.

Table S2. IC_{50} (μM) values of the investigated complexes toward the selected cancer cell lines for 24 h. Data are given as mean ± SD of 3 independent experiments (N=3).

	**MCF-7**	**Du-145**	**A549**	**PANC-1**	**HaCaT**
dibutylamine	>1000	>1000	>1000	>1000	>1000
diethylamine	>1000	>1000	>1000	>1000	>1000
**1**	458±11	203±19	620±9	426±7	646±20
**2**	355±13	185±12	569±11	386±6	556±13
**1a**	189±9	90±5	68±5	143±3	456±11
**2a**	125±15	69±6	79±6	106±5	559±25
**1b**	295±12	122±11	59±3	146±5	446±20
**2b**	226±20	102±6	81±4	126±6	545±13
**Cisplatin**	176±5	85±7	70±8	166±20	400±3

Table S3. IC_{50} (μM) values of the investigated complexes toward the selected cancer cell lines for 48 h. Data are given as mean ± SD of 3 independent experiments (N=3).

	**MCF-7**	**Du-145**	**A549**	**PANC-1**	**HaCaT**
dibutylamine	226±20	103±7	387±25	258±6	445±25
diethylamine	352±25	126±12	425±38	305±13	545±33
**1**	149±13	86±6	47±5	185±13	458±20
**2**	125±20	54±8	72±6	158±23	420±11
**1a**	184±6	85±9	45±4	204±29	446±26
**2a**	189±20	69±8	61±3	198±20	420±12
**1b**	352±25	126±12	425±38	305±13	545±33
**2b**	226±20	103±7	387±25	258±6	445±25
**Cisplatin**	151±6	81±8	66±9	131±7	362±4
